# Alnustone Ameliorates Metabolic Dysfunction‐Associated Steatotic Liver Disease by Facilitating Mitochondrial Fatty Acid β‐Oxidation via Targeting Calmodulin

**DOI:** 10.1002/advs.202411984

**Published:** 2025-06-05

**Authors:** Shourui Hu, Xiaofan Liang, Yiming Qin, Yuxuan Li, Yue Liu, Congcong Liu, Zongxuan Lin, Chunxuan Geng, Yanqi Xu, Daimin Wei, Yingying Qin, Han Zhao, Yuqing Zhang, Zi‐Jiang Chen

**Affiliations:** ^1^ State Key Laboratory of Reproductive Medicine and Offspring Health Center for Reproductive Medicine Institute of Women Children and Reproductive Health Shandong University Jinan Shandong 250012 China; ^2^ National Research Center for Assisted Reproductive Technology and Reproductive Genetics Shandong University Jinan Shandong 250012 China; ^3^ Key Laboratory of Reproductive Endocrinology (Shandong University) Ministry of Education Jinan Shandong 250012 China; ^4^ Shanghai Key Laboratory for Assisted Reproduction and Reproductive Genetics Shanghai 200135 China; ^5^ Department of Reproductive Medicine Ren Ji Hospital Shanghai Jiao Tong University School of Medicine Shanghai 200135 China

**Keywords:** alnustone, calmodulin, fatty acid β‐oxidation, metabolic dysfunction‐associated steatohepatitis, metabolic dysfunction‐associated steatotic liver disease

## Abstract

Metabolic dysfunction‐associated steatotic liver disease (MASLD), including its more severe manifestation metabolic dysfunction‐associated steatohepatitis (MASH), poses global public health threats with limited therapeutics. Here, the role of alnustone is explored, a natural compound derived from the traditional Chinese herb *Alpinia katsumadai* Hayata, in the treatment of MASLD and MASH. It is shown that alnustone administration potently reduces serum triacylglycerol levels, reverses liver steatosis, and alleviates insulin resistance in both male and female MASLD mice. It also effectively ameliorates established fibrosis in MASH mice without any side effects. Mechanistically, hepatic lipidome profiling and energy metabolic assays reveal that alnustone facilitates mitochondrial fatty acid β‐oxidation. Employing limited proteolysis‐mass spectrometry (LiP‐SMap) and further validation, calmodulin is identified as a direct molecular target of alnustone. Alnustone interacts with the Ca^2+^‐binding site of calmodulin, leading to increased cytosolic and mitochondrial Ca^2+^ levels and enhanced mitochondrial function, whereas liver‐specific calmodulin knockdown abrogates alnustone's therapeutic effects. Moreover, calmodulin is downregulated in human livers of patients with MASLD and MASH, and is genetically associated with reduced MASLD risk. These findings establish alnustone as a promising natural compound and highlight calmodulin as a target for treating MASLD.

## Graphical abstract

This study identifies alnustone, a natural compound from Alpinia katsumadai, as a potent therapeutic agent for MASLD and MASH. Alnustone enhances mitochondrial fatty acid β‐oxidation by directly targeting calmodulin, improving liver steatosis, fibrosis, and insulin resistance in vivo. These findings offer a promising therapeutic strategy and highlight calmodulin as a novel target for metabolic liver disease.

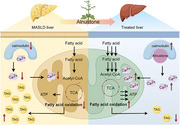

## 1 Introduction

With the dramatic shifts in modern people's dietary habits and lifestyle choices, metabolic dysfunction‐associated steatotic liver disease (MASLD) has become the most prevalent chronic liver disease and a worldwide healthcare problem.^[^ ^1^ ^]^ This spectrum of liver disorders, which ranges from simple steatosis to steatohepatitis, affects approximately 30% of the global population.^[^ ^2^ ^]^ Metabolic dysfunction‐associated steatohepatitis (MASH), an aggressive form of MASLD,^[^ ^3^ ^]^ further increases risk of progressing to advanced fibrosis, cirrhosis, hepatocellular carcinoma, and eventually liver‐related mortality.^[^ ^4^, ^5^ ^]^ Patients with MASLD also face increased risks for major adverse cardiovascular events, type 2 diabetes, and chronic kidney disease.^[^ ^6^ ^]^ Indeed, MASLD is emerging as the leading cause of liver failure and liver transplantation in the coming decades.^[^ ^7^, ^8^ ^]^ In the United States alone, the annual medical costs attributed to MASLD have escalated to $103 billion, affecting 64 million people.^[^ ^9^ ^]^ MASLD has also emerged as a significant public health concern in China, warranting increased attention. Recent studies have shown that the prevalence of MASLD in urban Chinese populations is as high as 28.77%,^[^ ^10^ ^]^ indicating a substantial burden of this metabolic liver disorder. With its increasing prevalence and severe impacts, MASLD continues to impose a heavy health and social‐economic burden worldwide.

The development of MASLD is characterized by a disruption in the liver's energy metabolic balance: an overload of carbohydrates and lipids exceeds the liver's capacity to oxidize or eliminate them, resulting in lipids accumulation in hepatocytes.^[^ ^4^ ^]^ The metabolic causes of hepatic steatosis include elevated hepatic de novo lipogenesis,^[^ ^11^ ^]^ defective esterification of fatty acids,^[^ ^12^ ^]^ impaired triacylglycerol export,^[^ ^13^ ^]^ decreased intrahepatic lipolysis,^[^ ^14^ ^]^ and reduced hepatic mitochondrial/peroxisomal β‐oxidation.^[^ ^15^ ^]^ Despite considerable advances in identifying potential therapeutic targets and developing innovative drugs over recent years, Resmetirom remains the only drug that has been approved by FDA specifically for MASLD and MASH.^[^ ^16^ ^]^ Discovery of medications that are both effective and safe, capable of reducing lipid accumulation and further reversing the progression of liver fibrosis, is urgently needed.

Alnustone (4(E)‐,6(E)‐1,7‐Diphenyl‐hepta‐4,6‐dien‐3‐one), a diarylheptanoid with a typical aryl‐C7‐aryl chemical structure, is an active ingredient in traditional Chinese herb *Alpinia katsumadai* Hayata.^[^ ^17^ ^]^ It was originally derived from the male flower of *Alnus pendula* and mainly isolated from the rhizomes of Curcuma *xanthorrhiza Roxb* and seeds of *Alpinia katsumadai* Hayata.^[^ ^18^, ^19^, ^20^, ^21^ ^]^ Previously, alnustone has demonstrated a range of biological activities including anti‐inflammatory, antibacterial, and anticancer properties.^[^ ^18^, ^22^, ^23^ ^]^ Among diarylheptanoids, curcumin has been extensively studied and noted for its therapeutic potentials in MASLD and associated metabolic disorders,^[^ ^24^ ^]^ particularly in improving lipid profiles and insulin sensitivity in patients with MASLD.^[^ ^25^ ^]^ Despite these insights, the therapeutic effects of alnustone in MASLD and MASH have never been explored.

In this study, we investigated the role of alnustone in ameliorating hepatic steatosis across various MASLD/MASH models. We identified calmodulin/Ca^2+^ signaling pathway as the critical mechanism through which alnustone enhanced mitochondrial fatty acid β‐oxidation. This discovery positions alnustone as a promising natural therapeutic agent for the treatment of MASLD/MASH by targeting calmodulin.

## 2 Results

### 2.1 Alnustone Attenuates HFD‐Induced Hepatic Steatosis and Insulin Resistance in Both Male and Female Mice

**Figure 1:**
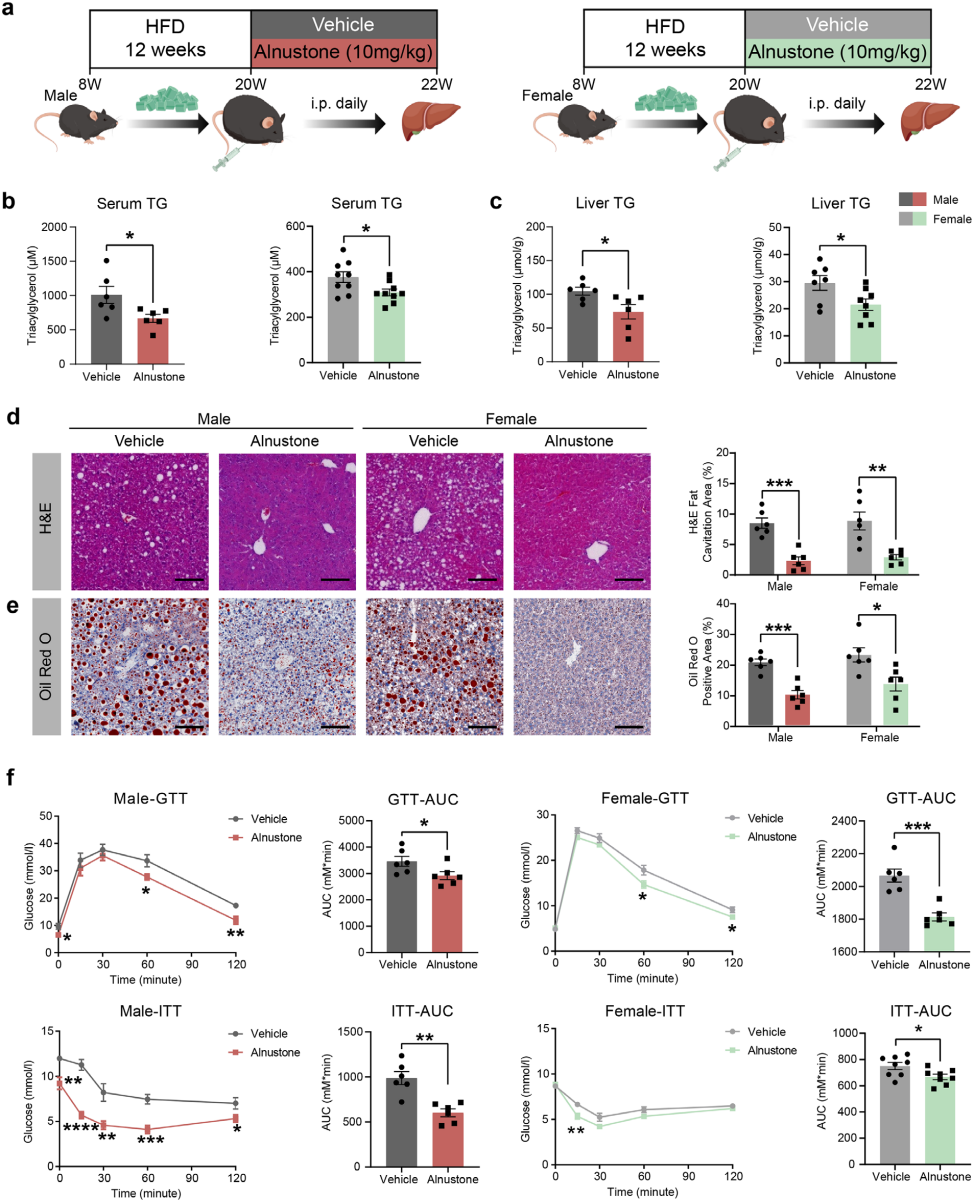
Alnustone alleviates hepatic steatosis and insulin resistance in male and female MASLD mice induced by high‐fat diet. a) Schematic illustration of intraperitoneal alnustone injection to MASLD mice induced by HFD. b) Serum triacylglycerol levels of male (n = 6) and female (n = 9) mice fed with HFD and administrated with vehicle/alnustone for 2 weeks. c) Hepatic triacylglycerol levels of male (n = 6) and female (vehicle: n = 7, alnustone: n = 8) mice fed with HFD and administrated with vehicle/alnustone for 2 weeks. Triacylglycerol contents were normalized by hepatic protein levels. d‐e) H&E (d) and Oil Red O (e) staining were performed in liver sections from vehicle/alnustone‐treated mice fed with HFD. H&E fat cavitation area, ORO staining area were quantitatively compared. Scale bar: 100 µm. (n = 6). f) Glucose tolerance test and insulin tolerance test were performed on male (n = 6) and female (n = 6‐8) mice administrated with alnustone or vehicle for 1 weeks and area under curve (AUC) was calculated and compared. Data are presented as mean ± SEM. *P < 0.05, **P < 0.01, ***P < 0.001, ****P < 0.0001; significance is assessed by two‐tailed unpaired Student's *t* test.

To explore the effects of alnustone on MASLD, both male and female mice were subjected to a high‐fat diet (HFD) feeding for a duration of 12 weeks to induce massive hepatic steatosis. Subsequently, a dosage of 10 mg kg^−1^ alnustone was administered intraperitoneally every‐day for a period of 2 weeks (**Figure** **1**a). In comparison to the HFD control mice, alnustone‐treated mice exhibited significantly diminished serum triacylglycerol levels, without altering total cholesterol levels (Figure 1b; Figure S1a, Supporting Information). To assess lipid accumulation in the liver, we quantified the liver triacylglycerol contents. Hepatic triacylglycerol decreased by 29% and 27% in male and female mice respectively after alnustone treatment (Figure 1c), along with significantly decreased liver cholesterol contents (Figure S1b, Supporting Information). Hematoxylin and eosin (H&E) and Oil Red O staining revealed that both male and female HFD mice displayed remarkably reduced lipid droplets accumulation in hepatocytes after alnustone treatment (Figure 1d,e), which is consistent with the results of liver triacylglycerol contents.

MASLD is usually associated with insulin resistance.^[^ ^26^ ^]^ We also examined glucose homeostasis through glucose tolerance test (GTT) and insulin tolerance test (ITT) assays. As early as one week after alnustone administration, alnustone‐treated mice showed significantly lower blood glucose levels and improved insulin sensitivity compared with controls during the tolerance tests, suggesting benefits of alnustone against MASLD pathogenesis (Figure 1f). Female mice displayed similar favorable phenotypes as male mice (Figure 1f).

To detect the impact of alnustone on the basic physiological functions of mice, we examined the effects of alnustone on body weight, liver function, and histopathology. Our results revealed that alnustone did not exert any significant influence on body weight and liver weight compared with the control group (Figure S1c,d, Supporting Information). Additionally, a thorough comparison of liver function tests and histological assessments revealed no toxic effects in mice treated with alnustone (Figure S1e,f (Supporting Information) and Figure 1d), demonstrating that alnustone treatment is both effective and safe for mice.

We also assessed the therapeutic effects of alnustone in a genetically induced MASLD mouse model – leptin receptor‐deficient (*db*/*db*) mice. Alnustone‐treated *db*/*db* mice exhibited significantly decreased serum triacylglycerol levels and hepatic triacylglycerol contents (Figure S2a, Supporting Information). In addition, alnustone treatment markedly reduced hepatic lipid accumulation as indicated by the H&E and Oil Red O staining (Figure S2b, Supporting Information), without any differences in total cholesterol levels, body weight, or liver weight (Figure S2c‐e, Supporting Information). These results collectively suggested that alnustone administration could effectively decrease circulating triacylglycerol levels, reverse hepatic lipid deposition, improve glucose and insulin intolerance in MASLD mice.

### 2.2 Oral Administration of Alnustone Effectively Alleviates HFD‐Induced MASLD

**Figure 2:**
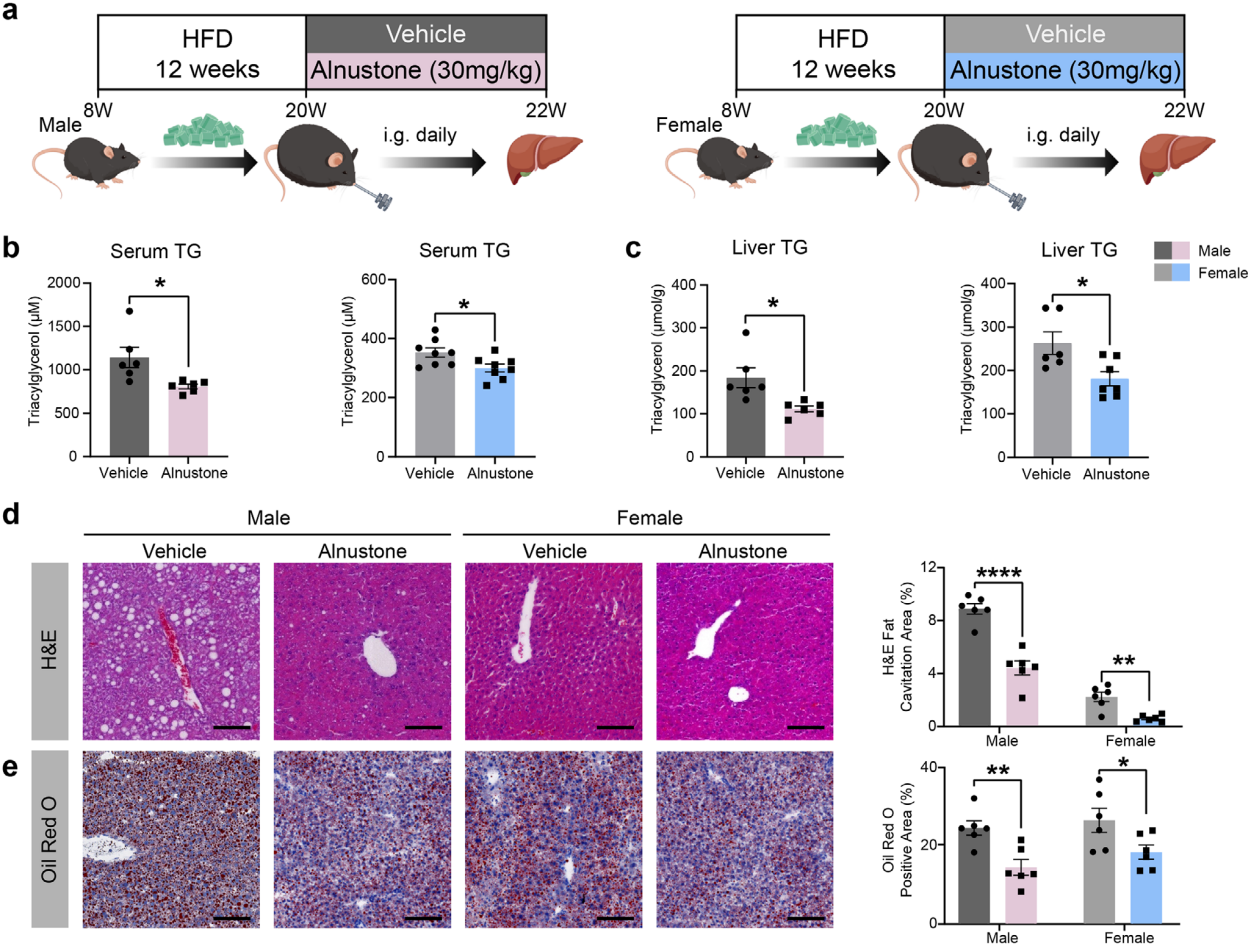
Oral administration of alnustone effectively ameliorates hepatic steatosis in HFD‐induced MASLD mice. a) Schematic illustration of oral alnustone administration to MASLD mice induced by HFD. b) Serum triacylglycerol levels of male (n = 6) and female (n = 8) mice fed with HFD and administrated with vehicle/alnustone for 2 weeks. c) Hepatic triacylglycerol levels of male (n = 6) and female (n = 6‐7) mice fed with HFD and administrated with vehicle/alnustone for 2 weeks. Triacylglycerol contents were normalized by hepatic protein levels. d‐e) H&E (d) and Oil Red O (e) staining were performed in liver sections from vehicle/alnustone‐treated mice fed with HFD. H&E fat cavitation area, ORO staining area were quantitatively compared. Scale bar: 100 µm. (n = 6). Data are presented as mean ± SEM. *P < 0.05, **P < 0.01, ****P < 0.0001; significance is assessed by two‐tailed unpaired Student's *t* test.

Considering that medications are usually delivered orally in clinical practice, we investigated whether oral administration of alnustone could alleviate MASLD. HFD‐induced MASLD mice were administered with 30 mg kg^−1^ alnustone or vehicle by oral gavage every‐day for 2 weeks (**Figure** **2**a). Consistent with previous results, serum triacylglycerol and hepatic triacylglycerol levels were significantly reduced by alnustone in both male and female mice (Figure 2b,c). Liver histological assessments and Oil Red O staining further confirmed reduced hepatic steatosis in alnustone‐treated mice (Figure 2d,e). While body weight, liver weight, and cholesterol levels were not changed (Figure S3a‐d, Supporting Information), the glucose intolerance and insulin resistance of HFD mice were considerably improved by oral alnustone administration (Figure S3e,f, Supporting Information). These findings demonstrated that both intraperitoneal injection and oral administration of alnustone can effectively alleviate hepatic steatosis and related metabolic dysfunctions.

### 2.3 Alnustone Ameliorates MCD‐Induced MASH in Mice

**Figure 3:**
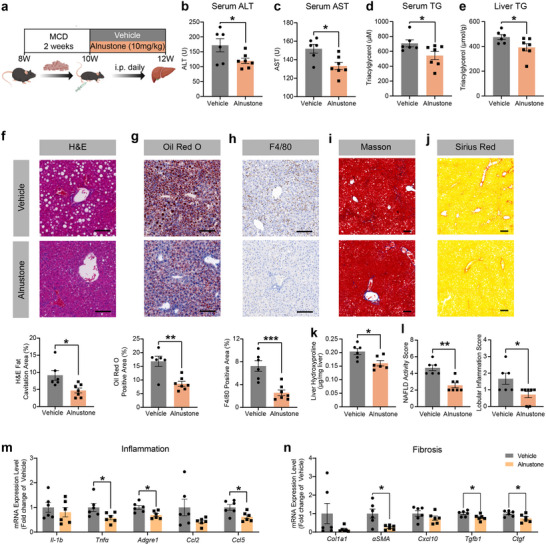
Alnustone alleviates hepatic steatosis, inflammation, and fibrosis in MCD‐induced MASH mice. a) Schematic illustration of intraperitoneal alnustone injection to MASH mice induced by MCD. b) Serum ALT levels of mice fed with MCD diets and administrated with vehicle/alnustone for 2 weeks. (vehicle: n = 6, alnustone: n = 7). c) Serum AST levels of mice fed with MCD diets and administrated with vehicle/alnustone for 2 weeks. (vehicle: n = 6, alnustone: n = 7). d) Serum triacylglycerol levels of mice fed with MCD diets and administrated with vehicle/alnustone for 2 weeks. (vehicle: n = 6, alnustone: n = 7). e) Hepatic triacylglycerol levels of mice fed with MCD diets and administrated with vehicle/alnustone for 2 weeks. Triacylglycerol contents were normalized by hepatic protein levels. (vehicle: n = 6, alnustone: n = 7). f‐j) H&E (f), Oil Red O (g), F4/80 (h), Masson (i), and Sirius red (j) staining were performed in liver sections from vehicle/alnustone‐treated mice fed with MCD diet. H&E fat cavitation area, ORO staining area, F4/80 staining area, Masson staining area, and Sirius red staining area were quantitatively compared. Scale bar: 100 µm. (vehicle: n = 6, alnustone: n = 7). k) Liver hydroxyproline content of mice fed with MCD diets and administrated with vehicle/alnustone for 2 weeks. (n = 6). l) NAFLD activity score and inflammation score were evaluated by comparing scores from liver sections. (vehicle: n = 6, alnustone: n = 7). m‐n) RNA was extracted from liver tissues of MCD diets‐induced MASH mice administrated with alnustone or vehicle and expression of inflammation (m) and fibrosis‐related (n) genes was determined by RT‐qPCR with β‐actin as an internal control. (n = 6). Data are presented as mean ± SEM. *P < 0.05, **P < 0.01, ***P < 0.001; significance is assessed by two‐tailed unpaired Student's *t* test or rank sum test.

Currently, the limited number of approved therapies for MASH is mainly attributed to the difficulty of developing effective agents to reverse fibrosis. To investigate the efficacy of alnustone in MASH, we administered a methionine‐ and choline‐deficient diet (MCD) to wildtype mice to develop a model of advanced MASH with a severe steatosis and fibrosis, as previously reported to recapitulate the progression of human MASH.^[^ ^27^, ^28^ ^]^ Male mice were fed with MCD to establish MASH, and were then randomized to 10 mg kg^−1^ alnustone or vehicle intraperitoneally along with continued MCD feeding for 2 weeks (**Figure** **3**a). After MCD feeding, MASH control mice displayed pronounced liver injury and dyslipidemia (Figure S4a‐c and Table S1, Supporting Information). Consistent with the above findings in HFD‐induced MASLD mice, alnustone significantly decreased serum triacylglycerol, alanine aminotransferase (ALT), and aspartate aminotransferase (AST) levels in mice with MASH (Figure 3b‐d), supporting an effect on alleviating liver injury.

In these MCD mice, alnustone treatment led to marked improvements in liver steatosis and inflammation, including hepatic triacylglycerol content, fat cavitation area, Oil Red O positive area, and F4/80 positive area (Figure 3e‐h). To be noted, hepatic fibrosis as indicated by Masson and Sirius red staining in alnustone‐treated MCD mice were significantly reversed (Figure 3i,j). Hepatic hydroxyproline measurement showed that the collagen content was significantly reduced after alnustone treatment (Figure 3k), further demonstrating the efficacy of alnustone at ameliorating fibrosis. MASH mice exhibited increased histological scoring of liver steatosis and inflammation, resulting in a higher NAFLD activity score (NAS) with severe fibrosis. Encouragingly, alnustone administration substantially reduced hepatic triacylglycerol level, the size and abundance of lipid droplets, the number of inflammatory lesions, as well as NAS levels (Figure 3l). In line with aforementioned result, marker genes related to pro‐inflammatory response (*Tnfα*, *Adgre1*, and *Ccl5*) were significantly downregulated by alnustone (Figure 3m). Furthermore, the expression levels of pro‐fibrosis genes (*α‐SMA*, *Tgfb1*, and *Ctgf*) were significantly decreased in the livers of alnustone‐treated mice (Figure 3n). Above results proved that alnustone treatment effectively ameliorates hepatic steatosis and prevents the progression of inflammation and fibrosis in MASH mice.

To benchmark the efficacy of alnustone against existing drugs, we compared the therapeutic effects of alnustone with Farnesoid X‐receptor (FXR) agonist obeticholic acid (OCA), a currently used classical drug for clinical treatment of MASLD and is being tested in phase III clinical trials in patients with MASH.^[^ ^29^ ^]^ OCA was administered with the same dose of alnustone in MCD‐induced MASH mice. Comparisons of the main characteristics of MASH revealed that alnustone potently decreased serum and hepatic triacylglycerol as well as ALT and AST levels, whereas OCA only significantly reduced hepatic triacylglycerol content in MASH mice (Figure S4a‐d and Table S1, Supporting Information). Alnustone exhibited superior efficacy over OCA in terms of reducing serum ALT and AST levels (Figure S4a,b and Table S1, Supporting Information). Moreover, alnustone exerted more pronounced effects on ameliorating hepatic lipid droplets accumulation and liver fibrosis (Figure S4e‐h, Supporting Information). These data demonstrate that alnustone showed superior therapeutic effects over the FXR agonist OCA on MASH therapy.

### 2.4 Alnustone Ameliorates AMLN‐Induced MASH in Mice

**Figure 4:**
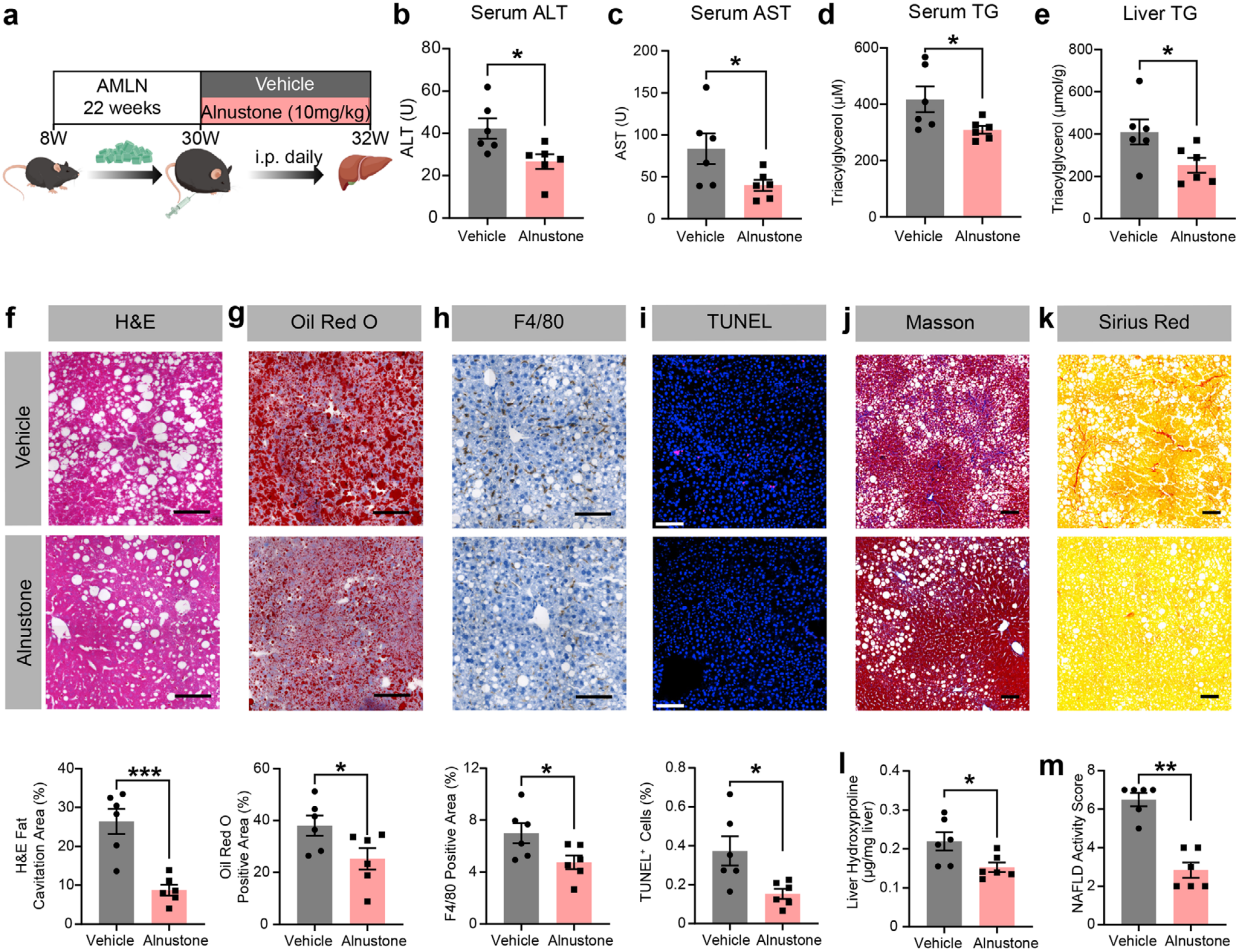
Alnustone alleviates hepatic steatosis, inflammation, and fibrosis in AMLN‐induced MASH mice. a) Schematic illustration of intraperitoneal alnustone injection to MASH mice induced by AMLN. b) Serum ALT levels of mice fed with AMLN diets and administrated with vehicle/alnustone for 2 weeks. (n = 6). c) Serum AST levels of mice fed with AMLN diets and administrated with vehicle/alnustone for 2 weeks. (n = 6). d) Serum triacylglycerol levels of mice fed with AMLN diets and administrated with vehicle/alnustone for 2 weeks. (n = 6). e) Hepatic triacylglycerol levels of mice fed with AMLN diets and administrated with vehicle/alnustone for 2 weeks. Triacylglycerol contents were normalized by hepatic protein levels. (n = 6). f‐k) H&E (f), Oil Red O (g), F4/80 (h), TUNEL (i), Masson (j), and Sirius red (k) staining were performed in liver sections from vehicle/alnustone‐treated mice fed with AMLN diet. H&E fat cavitation area, ORO staining area, F4/80 staining area, TUNEL positive cell percentage, Masson staining area, and Sirius red staining area were quantitatively compared. Scale bar: 100 µm. (n = 6). l) Liver hydroxyproline content of mice fed with AMLN diets and administrated with vehicle/alnustone for 2 weeks. (n = 6). m) NAFLD activity score was evaluated by comparing scores from liver sections. (n = 6). Data are presented as mean ± SEM. *P < 0.05, **P < 0.01, ***P < 0.001; significance is assessed by two‐tailed unpaired Student's *t* test or rank sum test.

To rigorously evaluate alnustone's therapeutic potential in MASH, we expanded our investigation to a high‐fat, high‐fructose, high‐cholesterol (AMLN) diet model. Administration of alnustone significantly attenuated hepatic injury, as evidenced by reduced serum ALT/AST levels (**Figure** **4**a‐c). Concomitantly, alnustone decreased systemic and hepatic triglyceride accumulation (Figure 4d,e), with histopathological analyses revealing a significant reduction in lipid droplet size by H&E and Oil Red O staining (Figure 4f,g). Critically, F4/80 immunohistochemistry and flow cytometry demonstrated that alnustone decreased hepatic monocyte and macrophage infiltration and rescued the depletion of Kupffer cells (Figure 4h; Figure S5a‐d, Supporting Information), indicative of suppressed inflammatory responses. TUNEL staining showed the apoptotic hepatocytes were significantly reduced in alnustone‐treated mice, suggesting alnustone plays protective roles in the hepatic injury and cell death of MASH (Figure 4i).

To be noted, hepatic fibrosis remodeling was remarkably mitigated by alnustone, with Sirius Red and Masson staining showing a reduction in collagen deposition, corroborated by hydroxyproline assays (Figure 4j‐l). Consistent with observations in the MCD mice, alnustone ameliorated hepatic steatosis and inflammation in AMLN‐fed mice, as evidenced by a significant reduction in the NAS (Figure 4m). Collectively, alnustone exhibits consistent therapeutic benefits across mechanistically distinct preclinical MASH models.

### 2.5 Alnustone Protects Against Palmitic Acid‐Induced Steatosis in Mouse and Human Hepatocytes

**Figure 5:**
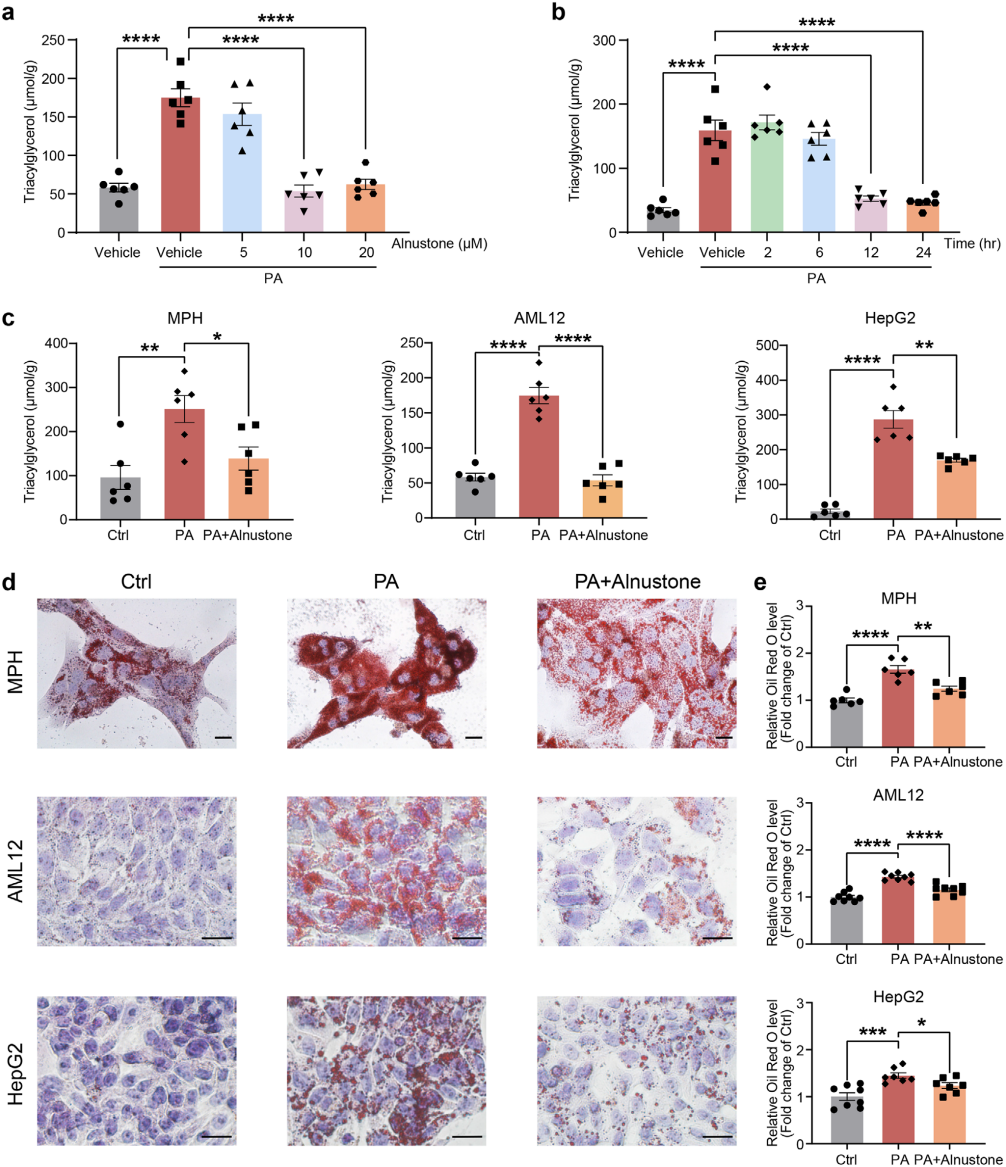
Alnustone protects mouse and human hepatocytes from lipid accumulation. a) AML12 cells were treated with 0.2 mM palmitic acid (PA) in the presence of vehicle or alnustone at the dose of 5, 10, 20 µM. After 24 h, cellular triacylglycerol levels were assayed. (n = 6). b) AML12 cells were treated with 0.2 mM palmitic acid for 24 h, and then alnustone was added to the medium for 2 h, 6 h, 12 h, 24 h. Cellular triacylglycerol levels were then assayed. (n = 6). c) Analysis of cellular triacylglycerol levels in mouse primary hepatocytes, AML12, and HepG2 cells treated with 0.2 mM palmitic acid and 10 µM alnustone for 24 h. (n = 6). d‐e) Cells under the indicated conditions in (c) were stained with Oil Red O (d), and concentrations of oil red were extracted by isopropanol and quantified (e). Scale bar: 20 µm. (n = 6‐8). Data are presented as mean ± SEM. *P < 0.05, **P < 0.01, ***P < 0.001, ****P < 0.0001; significance is assessed by two‐tailed unpaired Student's *t* test and one‐way ANOVA with Tukey's multiple comparisons post‐hoc test.

To ascertain the direct impact of alnustone on hepatocellular fat contents, we first evaluated the concentration‐ and time‐dependent effects of alnustone on hepatocytes against lipotoxicity. To induce steatosis, a mouse hepatocyte cell line—AML12 cells were exposed to 0.2 mM palmitic acid for 24 h, and cells were treated with different concentrations of alnustone (5, 10, 20 µM). Alnustone dose‐dependently reduced palmitic acid‐induced lipid accumulation in AML12 cells, showing a marked effect at the dose of 10 µM (**Figure** **5**a). Then AML12 cells exposed to palmitic acid were treated with 10 µM alnustone for various time (2, 6, 12, 24 h), which revealed that the lipid‐lowering effect of alnustone started to show at 12 h and persisted into 24 h after treatment (Figure 5b).

We then assessed the intracellular triacylglycerol contents using mouse primary hepatocytes, AML12 cells, and a human hepatocyte cell line—HepG2 cells. These cells were incubated with palmitic acid to induce steatosis and treated with 10 µM alnustone. As expected, alnustone significantly inhibited intracellular triacylglycerol contents in all three hepatocyte cell lines, without changing cellular total cholesterol contents (Figure 5c; Figure S6a, Supporting Information). Consistently, Oil Red O staining revealed alnustone dramatically declined the size and number of lipid droplets in palmitic acid‐induced hepatocytes (Figure 5d,e). Together, these results strongly support the direct potential of alnustone in the protection of both mouse and human hepatocytes from steatosis.

### 2.6 Alnustone Reduces Hepatic Glycerides Accumulation by Facilitating Mitochondrial Fatty Acid β‐Oxidation

**Figure 6:**
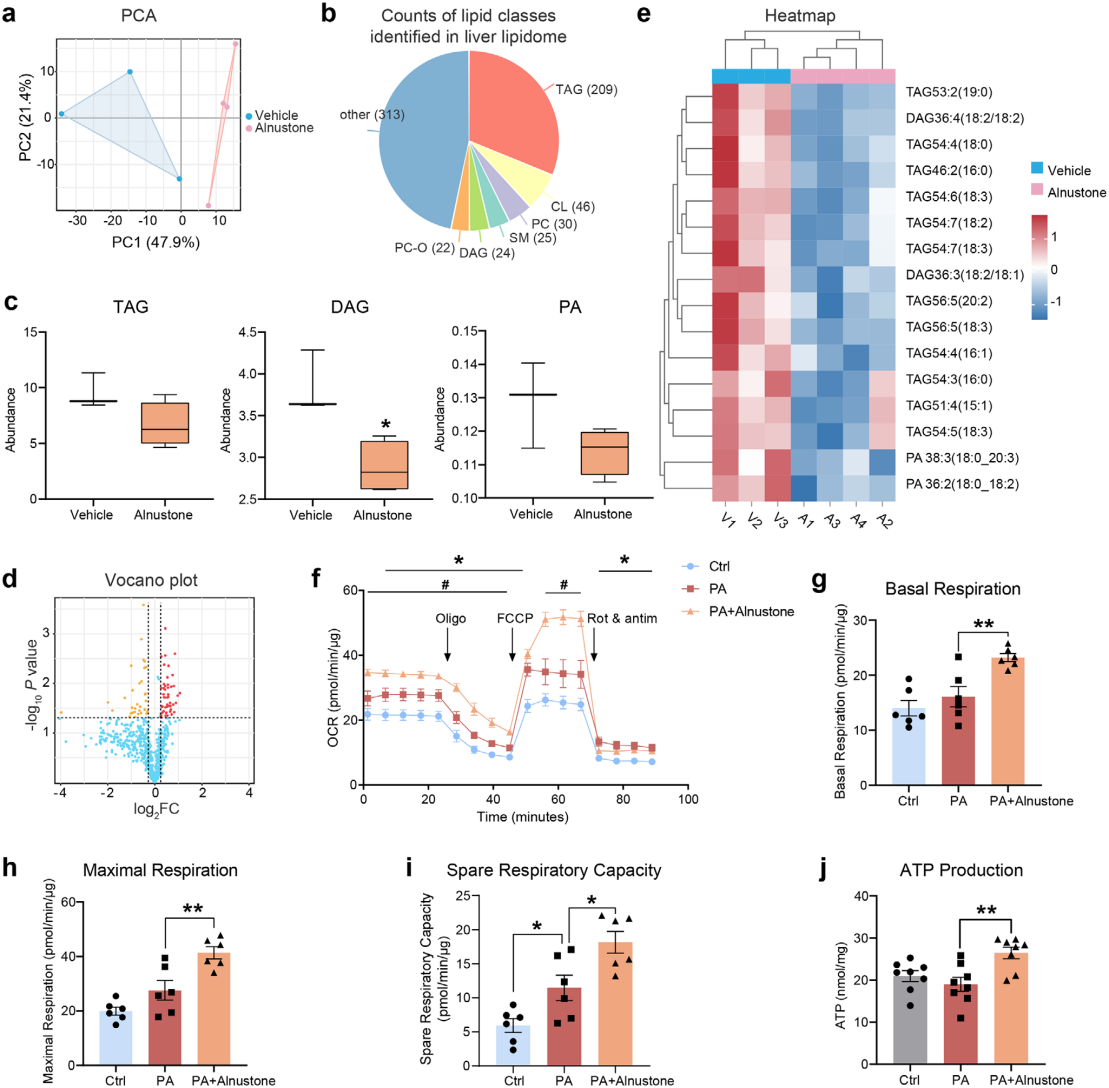
Alnustone reduces hepatic lipid metabolites by promoting mitochondrial fatty acid β‐oxidation. a) Principal component analysis (PCA) of liver lipidome from HFD‐induced mice treated with vehicle (n = 3) or alnustone (n = 4). b) Counts of lipid classes identified in liver lipidome. TAG: triacylglycerol; DAG: diacylglycerol; PA: palmitic acid. c) Relative abundance of the TAG, DAG, and PA class identified in liver lipidome. d) Volcano plot showing changes in lipid metabolites after alnustone treatment. e) Heatmap illustrating the effects of alnustone on individual TAG, DAG, and PA metabolites identified in liver lipidome. f‐i) Mitochondrial oxygen consumption rate (OCR) was measured by Seahorse assay in AML12 cells induced by 0.2 mM palmitic acid with or without 10 µM alnustone for 24 h (f). *, vehicle versus PA; #, PA versus PA+Alnustone. Basal respiration (g), maximal respiration (h), and spare respiratory capacity (i) were calculated. (n = 6). j) ATP content of AML12 cells induced by 0.2 mM palmitic acid with or without 10 µM alnustone for 24 h. (n = 8). Data are presented as mean ± SEM. *, #*P* < 0.05, **P < 0.01; significance is assessed by two‐tailed unpaired Student's *t* test.

To elucidate the potential mechanisms underlying the beneficial effect of alnustone in MASLD, we first dissected the comprehensive lipidomic signatures after alnustone treatment. Livers from vehicle‐ and alnustone‐treated HFD mice were subjected to lipidome profiling. The two groups were readily distinguishable by principal component analysis (**Figure** **6**a). A total of 34 classes of lipids were identified, among which triacylglycerol was the most abundant and largely changed lipid class (Figure 6b). Consistent with the lipid‐lowering phenotypes in alnustone‐treated mice, we found that the abundance of total triacylglycerol (TAG), diacylglycerol, and palmitic acid was greatly decreased (Figure 6c). In addition, a substantial amount of lipid metabolites was downregulated by alnustone treatment as shown in the volcano plot (Figure 6d). Further differential analysis showed that a variety of TAG and downstream metabolites were significantly decreased in alnustone‐treated livers, particularly the metabolites containing saturated fatty acid (Figure 6e).

As both de novo lipogenesis and fatty acid β‐oxidation mainly occur in mitochondria, we further sought to evaluate the mitochondrial mechanisms underlying alnustone‐improved lipid metabolism. For this purpose, mitochondrial respiratory functions were directly assessed by Seahorse analysis in AML12 hepatocytes. Measurements of oxygen consumption rate with the availability of palmitic acid revealed that mitochondrial fatty acid β‐oxidation was significantly facilitated by alnustone (Figure 6f). Moreover, the basal respiration, maximal respiration, spare respiratory capacity, and ATP levels were all significantly enhanced in palmitic acid‐induced hepatocytes treated with alnustone. These data indicate that alnustone increases lipid expenditure by promoting mitochondrial fatty acid β‐oxidation in hepatocytes (Figure 6g‐j).

### 2.7 Alnustone Directly Binds to Calmodulin Leading to Increased Calcium Levels

**Figure 7:**
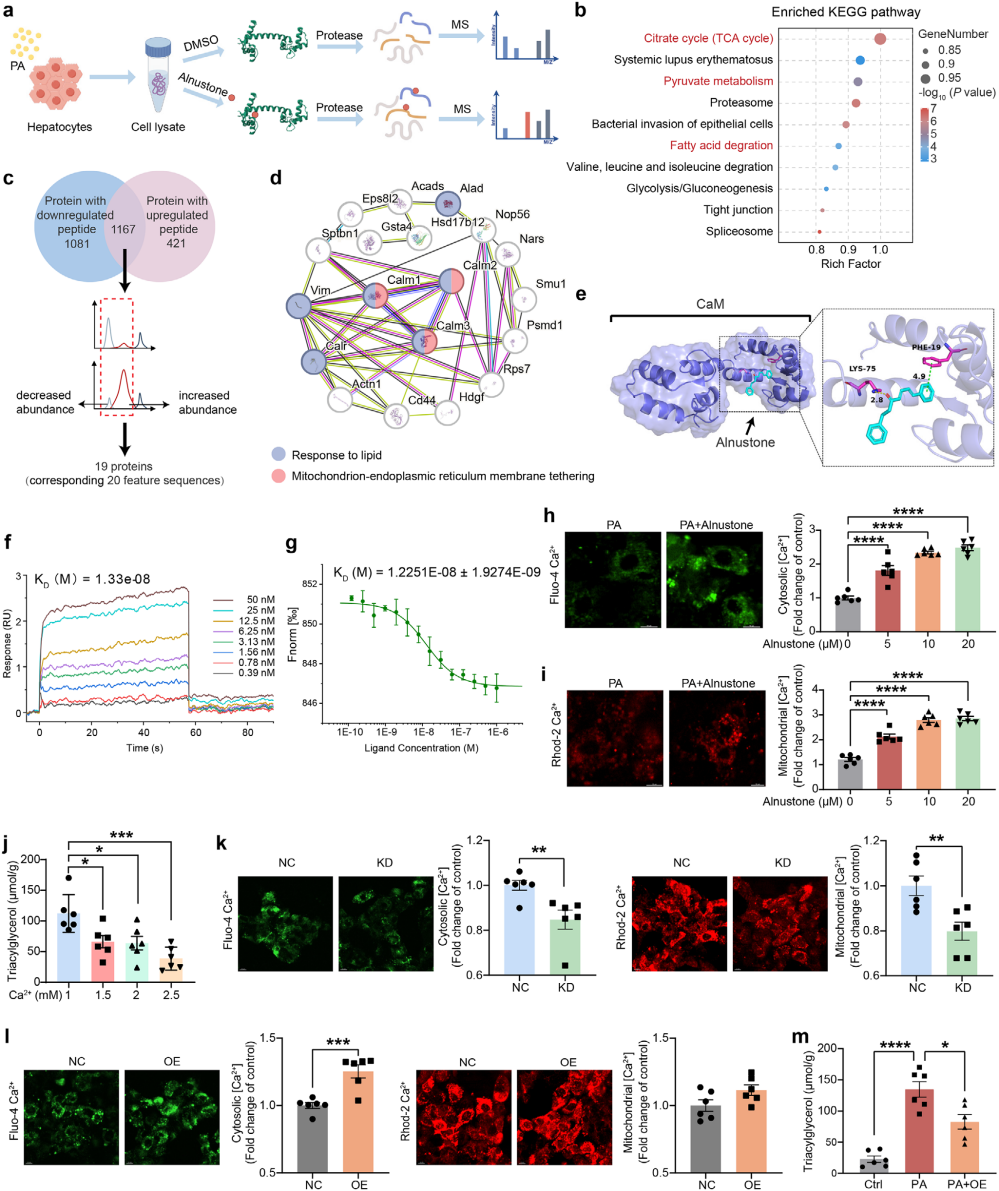
Alnustone directly binds to calmodulin resulting in increased cytosolic and mitochondrial Ca^2+^ levels. a) Flow chart depicting the LiP‐SMap assay. Freshly prepared AML12 whole‐cell lysates after exposed to palmitic acid for 24 h were treated with or without alnustone followed by proteinase K digestion and mass spectrometry (MS) analysis. The binding of alnustone prevents proteinase digestion, leading to the differential MS peptide profiling. b) KEGG enrichment of differentially proteins corresponding to differentially abundant peptides identified by LiP‐SMap. c) Schematic diagram illustrating the screening process of peptides corresponding to alnustone bound proteins. d) STRING network of the candidate targets of alnustone. Protein terms showing significant enrichment (*P* adj < 0.05) were identified and those with strength > 0.01 are ranked in figure. e) Representative images of autodocking for alnustone and CaM. The CaM protein is represented as a slate cartoon model, ligand is shown as a cyan stick, and their binding sites are shown as magentas stick structures. The hydrogen bond, ionic interactions, and hydrophobic interactions are depicted as yellow, magentas and green dashed lines, respectively. f) SPR analysis of the interactions between calmodulin and alnustone. g) Binding affinity of alnustone to calmodulin was detected by MST. h) Representative images of Fluo‐4 Ca^2+^ fluorescence after treatment with vehicle or 10 µM alnustone in the presence of palmitic acid. Scale bar: 10 µm. Fluorescence intensity of the calcium indicator Fluo‐4/AM was detected with fluorescence microscopy. (n = 6). i) Representative images of Rhod‐2 Ca^2+^ fluorescence after treatment with vehicle or 10 µM alnustone in the presence of palmitic acid. Scale bar: 10 µm. Fluorescence intensity of the calcium indicator Rhod‐2/AM was detected with fluorescence microscopy. (n = 6). j) Analysis of cellular triacylglycerol levels in AML12 cells treated with 1 µM, 1.5 µM, 2 µM, and 2.5 µM CaCl_2_. (n = 6). k) Representative images of Fluo‐4 Ca^2+^ and Rhod‐2 Ca^2+^ fluorescence after treatment with or without *Calm1‐3* knockdown in the presence of palmitic acid. Scale bar: 10 µm. Fluorescence intensity was detected with fluorescence microscopy. (n = 6). l) Representative images of Fluo‐4 Ca^2+^ and Rhod‐2 Ca^2+^ fluorescence after treatment with or without *Calm1* overexpression in the presence of palmitic acid. Scale bar: 10 µm. Fluorescence intensity was detected with fluorescence microscopy. (n = 6). m) Analysis of cellular triacylglycerol levels in AML12 cells with or without *Calm1* overexpression in the presence of palmitic acid. (n = 6). Data are presented as mean ± SEM. *P < 0.05, **P < 0.01, ***P < 0.001, ****P < 0.0001; significance is assessed by two‐tailed unpaired Student's *t* test and one‐way ANOVA with Tukey's multiple comparisons post‐hoc test.

To identify the molecular targets of alnustone, we employed LiP‐SMap to search for cellular proteins that could directly bind alnustone. In this workflow, palmitic acid‐induced AML12 cell lysates (three biological replicates per treatment) were incubated with alnustone or vehicle, followed by limited proteolysis with proteinase K, trypsin, and liquid chromatography‐tandem mass spectrometry (**Figure** **7**a). Through this approach, a total of 12 297 peptides annotated to 2669 (1588 up and 2248 down) significantly changed proteins were identified (Figure S7a, Supporting Information). Kyoto Encyclopedia of Genes and Genomes (KEGG) pathway enrichment analyses of these differentially abundant proteins demonstrated that mitochondrial pathways, including “Citrate cycle,” “Pyruvate metabolism,” and “Fatty acid degradation”, were significantly enriched (Figure 7b), which was in consistent with the enhanced mitochondrial function observed after alnustone treatment. Based on the methodology of LiP‐SMap,^[^ ^30^ ^]^ we first screened for 1167 proteins with both increased and decreased peptide identified after alnustone incubation. Then we mapped these peptides to the corresponding proteins to screen for peptides that were both up‐regulated and down‐regulated in the same or adjacent sequences, which could be ascribed to the binding of alnustone and therefore escaping proteolysis. Through this approach, a total of 19 proteins were identified as potential candidates (Figure 7c). STRING analysis of these candidates revealed marked enrichments in “Response to lipid” and “Mitochondrion‐endoplasmic reticulum membrane tethering” (Figure 7d). As shown in the network, CALM1, CALM2, and CALM3 are the core molecules.

CALM1‐3 encoded by three independent genes (*Calm1‐Calm3*) express the identical calmodulin (CaM) proteins,^[^ ^31^ ^]^ which possess intracellular Ca^2+^‐binding functions.^[^ ^32^ ^]^ To assess the exact binding site of alnustone on CaM, we carried out molecular docking. The derived binding mode showed that Lys75 and Phe19 residues of CaM, which locate exactly in the EF‐hand/Ca^2+^‐binding site of this protein,^[^ ^33^ ^]^ were involved in forming hydrogen bonds with alnustone (Figure 7e). The docking energy of the CaM‐alnustone complex was ‐7.0 kcal mol^−1^, indicating alnustone binds to CaM with high affinity. To validate the binding of alnustone to CaM in kinetic experiments, we performed surface plasmon resonance (SPR) with recombinant calmodulin protein, which was a widely recognized method for studying the dynamic properties between ligands and donors.^[^ ^34^ ^]^ As shown in the SPR result, the dissociation constant (Kd) of alnustone and calmodulin was 13 nM (Figure 7f), indicative of a strong interaction. Additionally, microscale thermophoresis (MST) assay further revealed calmodulin was readily bound by alnustone, with a Kd value of 12 nM (Figure 7g), indicating a robust interaction between alnustone and calmodulin. The above findings validate that calmodulin is a bona fide binding target of alnustone.

To evaluate whether alnustone binding to CaM contributes to altered Ca^2+^ signaling in hepatocytes, intracellular Ca^2+^ levels were assessed after alnustone treatment in the presence of palmitic acid‐induced steatosis. As shown in Figure 7h, cytosolic Ca^2+^ concentration was significantly elevated by alnustone in a dose‐dependent manner. Elevation in cytosolic Ca^2+^ is known to facilitate mitochondrial Ca^2+^ influx through the mitochondrial calcium uniporter.^[^ ^35^, ^36^ ^]^ Considering the enhanced mitochondrial activity by alnustone, we next detected mitochondrial Ca^2+^ concentration by using the mitochondrial calcium indicator Rhod‐2/AM, which revealed significantly increased mitochondrial Ca^2+^ levels after alnustone treatment (Figure 7i). Consistently, exogeneous Ca^2+^ treatment dose‐dependently decreased intracellular triglyceride content and lipid accumulation (Figure 7j; Figure S7b, Supporting Information), mimicking the therapeutic effects of alnustone.

To directly assess the role of CaM in the mobilization of Ca^2+^, we concurrently measured cytoplasmic and mitochondrial Ca^2+^ concentration following CaM knockdown or overexpression (Figure S7c,d, Supporting Information). The results showed that CaM knockdown via siRNA in hepatocytes led to significantly lower cytosolic and mitochondrial Ca^2+^ levels compared to controls (Figure 7k). This suggested that CaM is essential for maintaining normal Ca^2+^ homeostasis in hepatocytes. Conversely, overexpression of CaM in hepatocytes resulted in increased cytosolic and mitochondrial Ca^2+^ levels (Figure 7l), further supporting the role of CaM in regulating Ca^2+^ dynamics. As shown in Figure 7m and Figure S7e (Supporting Information), overexpression of CaM significantly ameliorates palmitate‐induced lipid accumulation in hepatocytes. These results further support that alnustone binding to CaM promotes its activity, leading to increased calcium influx and activation of mitochondrial function.

### 2.8 Calmodulin Mediates the Therapeutic Effects of Alnustone via Enhancing Ca^2+^‐Induced Mitochondrial Fatty Acid β‐Oxidation

**Figure 8:**
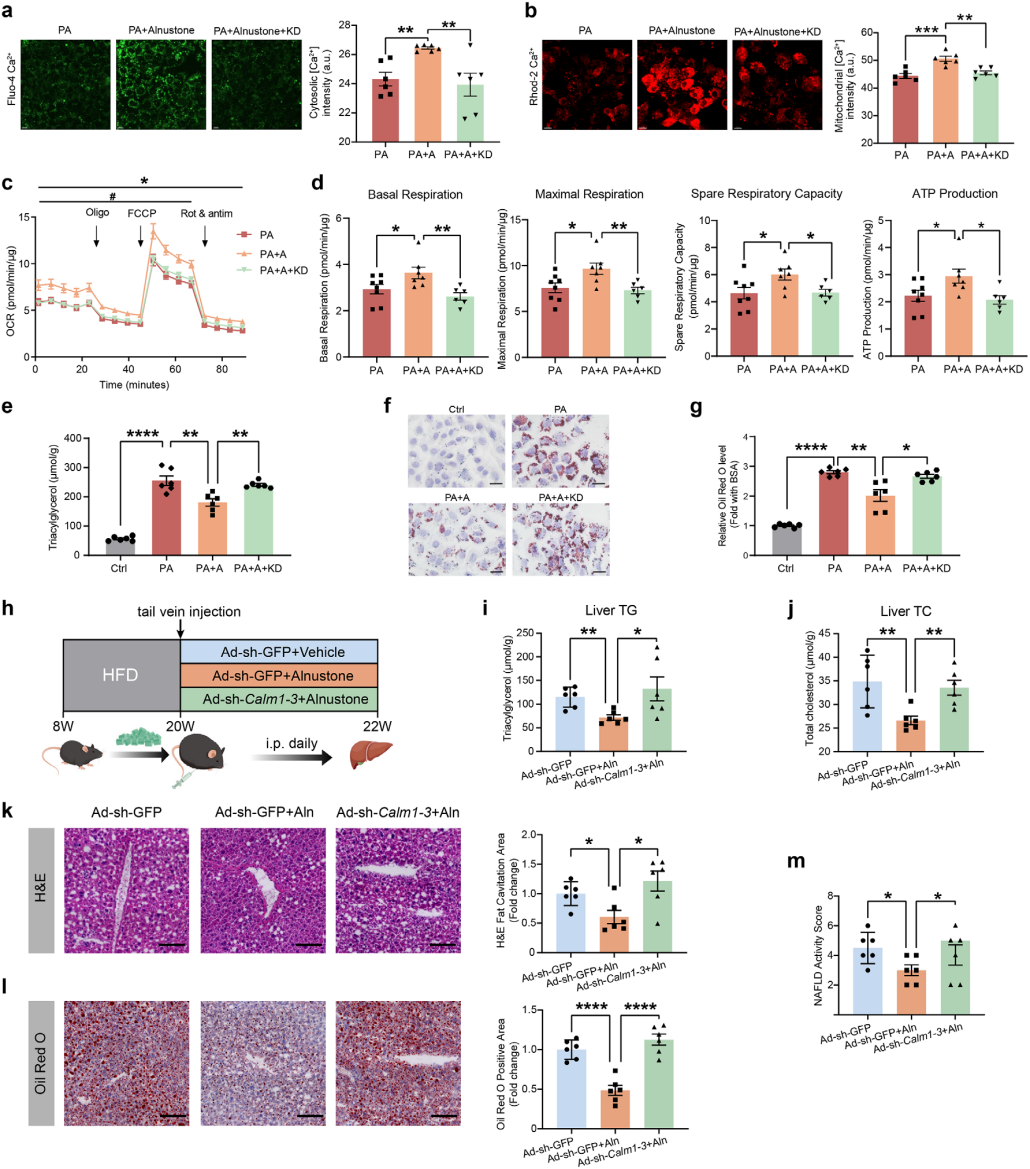
Calm1‐3 knockdown abrogates the therapeutic effects of alnustone on hepatic steatosis and mitochondrial fatty acid β‐oxidation. a) Representative images of Fluo‐4 Ca^2+^ fluorescence in AML12 cells treated with PA, PA with alnustone, or PA with alnustone after *Calm1*, *Calm2* and *Calm3* knockdown. Scale bar: 10 µm. Fluorescence intensity of the calcium indicator Fluo‐4/AM was detected with fluorescence microscopy. (n = 6). b) Representative images of Rhod‐2 Ca^2+^ fluorescence in AML12 cells treated with PA, PA with alnustone, or PA with alnustone after *Calm1*, *Calm2* and *Calm3* knockdown. Scale bar: 15 µm. Fluorescence intensity of the calcium indicator Rhod‐2/AM was detected with fluorescence microscopy. (n = 6). c‐d) Mitochondrial oxygen consumption rate (OCR) was measured by Seahorse assay in AML12 cells treated with PA, PA with alnustone, or PA with alnustone after *Calm1*, *Calm2*, and *Calm3* knockdown (c). *, PA versus PA+A; #, PA+A versus PA+A+KD. Basal respiration, maximal respiration, spare respiratory capacity, and ATP production were calculated (d). (n = 6‐8). e‐g) Analysis of cellular triacylglycerol levels in AML12 cells treated with vehicle, PA, PA with alnustone, or PA with alnustone after *Calm1*, *Calm2*, and *Calm3* knockdown. Cells under the indicated conditions in (e) were stained with Oil Red O (f), pictured using an inverted phase contrast microscope, and quantified concentrations of oil red extracted by isopropanol (g). Scale bar: 20 µm. h) Schematic overview for *Calm1*, *Calm2* and *Calm3* knockdown mice experiments. i‐j) Hepatic triacylglycerol levels (i) and total cholesterol levels (j) of HFD‐induced mice injected with Ad‐sh‐GFP or Ad‐sh‐*Calm1‐3* and administrated with vehicle/alnustone for 2 weeks. Triacylglycerol contents were normalized by hepatic protein levels. (n = 6). k‐l) H&E (k) and Oil Red O (l) staining were performed in liver sections from mice under the indicated conditions. Fat cavitation area and ORO staining area were quantitatively compared. Scale bar: 100 µm. m) NAS was compared by measuring scores from biopsy liver sections. (n = 6). Data are presented as mean ± SEM. *, #*P* < 0.05, **P < 0.01, ***P < 0.001, ****P < 0.0001; significance is assessed by two‐tailed unpaired Student's *t* test or rank sum test.

To verify whether CaM is required for alnustone's effects on mitochondrial and lipid metabolism, we concurrently knocked down *Calm1*, *Calm2*, and *Calm3* in hepatocytes and then treated with alnustone. Analyses of cellular Ca^2+^ concentrations showed that CaM knockdown markedly blocked alnustone's effects on elevating cytosolic and mitochondrial Ca^2+^ levels (**Figure** **8**a,b). In line with this result, Seahorse analysis showed that CaM knockdown remarkably abrogated alnustone‐promoted mitochondrial palmitic acid β‐oxidation in hepatocytes (Figure 8c). Alnustone‐induced mitochondrial basal respiration, maximal respiration, spare respiratory capacity, and ATP levels were returned to control levels after CaM knockdown (Figure 8d). Notably, CaM knockdown significantly compromised alnustone‐inhibited triacylglycerol accumulation in the presence of palmitic acid (Figure 8e‐g).

To validate the hepatic metabolic effects of CaM in vivo, we further knocked down liver CaM expression in MASLD mice by injecting adenovirus targeting *Calm1, Calm2*, and *Calm3*. HFD‐induced MASLD mice were injected with Ad‐sh‐*Calm1‐3* or Ad‐sh‐GFP control through tail vein (Figure 8h). The mRNA levels of *Calm1, Calm2*, and *Calm3* were specifically decreased in livers of mice injected with Ad‐sh‐*Calm1‐3* (Figure S7f, Supporting Information). Our results showed that alnustone's therapeutic effects on reducing hepatic triacylglycerol and cholesterol levels were significantly eliminated in liver‐specific CaM knockdown mice (Figure 8i,j). Moreover, the effects of alnustone on reducing hepatic steatosis and fibrosis were completely abrogated in hepatic CaM knockdown mice (Figure 8k‐m). Taken together, the above in vitro and in vivo results collectively demonstrate that CaM is a bona fide target of alnustone, which mediates the therapeutic effects of alnustone by facilitating Ca^2+^‐induced mitochondrial fatty acid β‐oxidation in hepatocytes.

### 2.9 Calmodulin is Downregulated in Human Livers of MASLD and Genetically Associated with Reduced Human MASLD Risk

**Figure 9:**
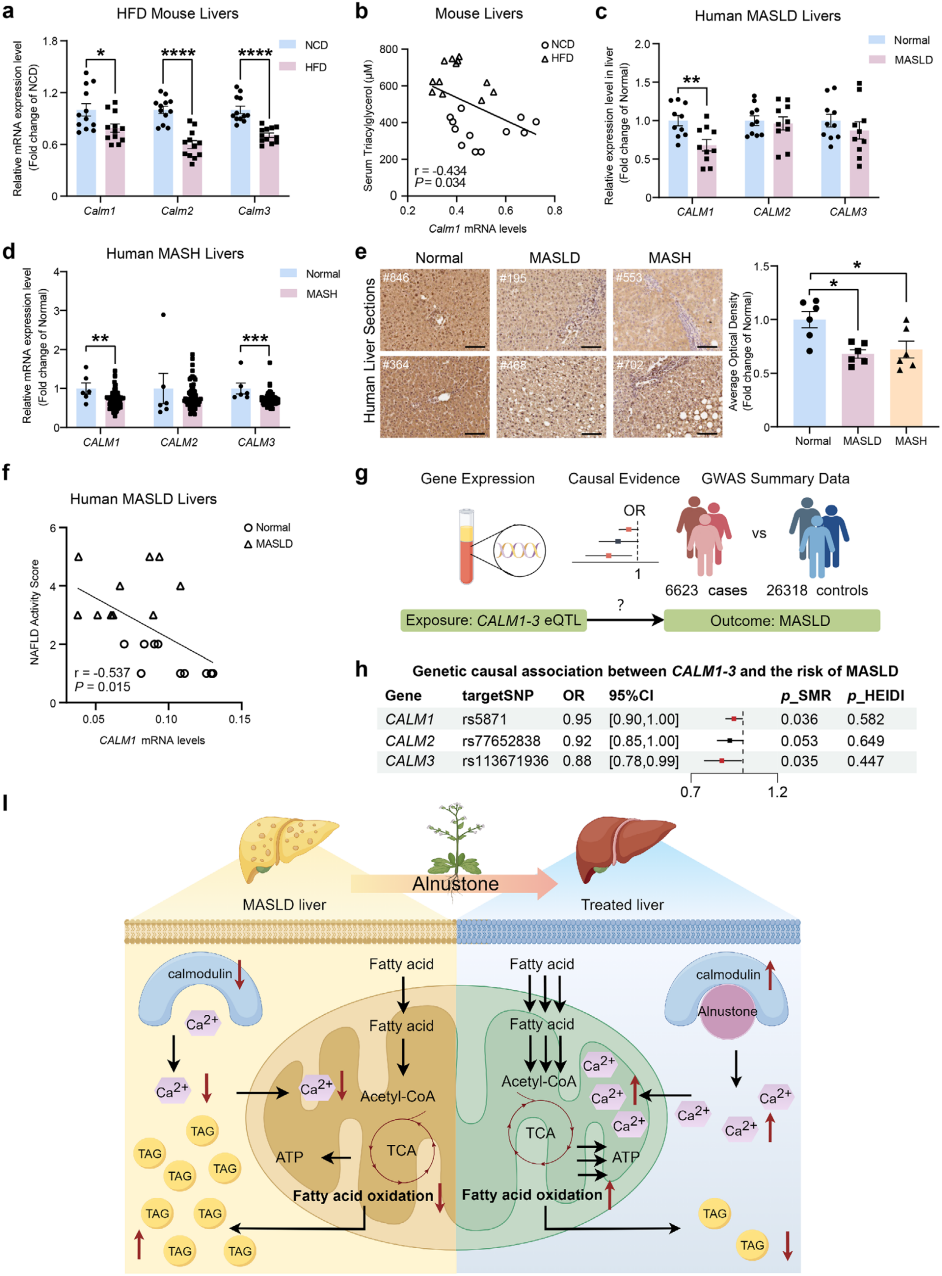
Calmodulin expression is decreased in human livers of MASLD and is genetically associated with reduced risk of human MASLD. a) Relative mRNA expressions of calmodulin in the livers of mice fed a normal chow diet (NCD) or HFD for 12weeks. (n = 12). b) Linear regression analysis of *Calm1* mRNA levels and serum triacylglycerol levels in the livers of NCD and HFD‐induced mice. (n = 12). c) Relative mRNA expressions of calmodulin in the livers of normal control subjects and MASLD patients. (n = 10). d) Relative mRNA expressions of calmodulin in the livers of normal control subjects and MASH patients from the GEO GSE164760 dataset. e) Representative immunohistochemistry images of human liver sections from normal control subjects, MASLD patients, and MASH patients. Scale bar: 100 µm. Average optical density was quantitatively compared. (n = 6). f) Linear regression analysis of *CALM1* mRNA levels and NAS in normal control subjects and MASLD patients. (n = 10). g) Schematic illustration of genetic causal association between *CALM1‐3* and the risk of MASLD. The expression quantitative trait loci (eQTL) of *CALM1‐3* were used as exposures and the summary GWAS data from 6623 MASLD patients and 26318 controls were employed as outcome. h) Genetic causal associations of *CALM1*, *CALM2*, and *CALM3* with human MASLD in summary data‐based Mendelian randomization analysis. SNP, single nucleotide polymorphisms; OR, odds ratio; CI, confidence interval; HEIDI, heterogeneity in the dependent instrument. i) Model depicting the role of alnustone in the treatment of MASLD: Alnustone increases cytosolic and mitochondrial Ca^2+^ concentration via binding to calmodulin, which in turn promotes mitochondrial fatty acid β‐oxidation, thereby contributing to lower hepatic triacylglycerol and ameliorating MASLD/MASH. The upward arrow denotes protein activated by binding to alnustone or augmentation of the corresponding process. Image was created by Figdraw. TCA: tricarboxylic acid cycle. Data are presented as mean ± SEM. *P < 0.05, **P < 0.01, ***P < 0.001, ****P < 0.0001; significance is assessed by two‐tailed unpaired Student's *t* test and one‐way ANOVA with Tukey's multiple comparisons post‐hoc test.

To investigate whether calmodulin is dysregulated in MASLD, we first assessed the mRNA expression levels of *Calm1*, *Calm2*, and *Calm3* in mouse models of MASLD, which were found to be significantly reduced in the livers of HFD mice (**Figure** **9**a). Supporting this observation, data from the Gene Expression Omnibus dataset (GSE109327) also demonstrated significantly lower *Calm2* mRNA levels in the livers from HFD mice (Figure S8a, Supporting Information). Moreover, we identified a negative correlation between hepatic *Calm1* mRNA expression and serum triacylglycerol levels in mice (Figure 9b). To validate these findings in clinic, we analyzed *CALM* expression in the liver samples from human subjects (Table S2, Supporting Information). Notably, we found that compared with normal livers, livers from patients with MASLD exhibited significantly lower mRNA expression levels of *CALM1* (Figure 9c). Reduced *CALM1* and *CALM3* mRNA expressions were further observed in the livers from patients with MASH (Figure 9d; Figure S8b,c, Supporting Information). Immunohistochemical analysis also showed reduced calmodulin protein abundance in the hepatocytes of patients with MASLD and MASH (Figure 9e). Linear regression analyses further confirmed *CALM1* expression levels were negatively correlated with NAS in human livers (Figure 9f). These data demonstrate that CaM is downregulated in livers of patients with MASLD, suggesting its critical role in the pathogenesis of human MASLD.

To determine the genetically causal role of calmodulin in human MASLD, we conducted summary‐data‐based Mendelian randomization (SMR) analysis to assess the causal associations between genetic variants linked to *CALM* gene expression levels and the risk of MASLD. For this purpose, the expression quantitative trait loci (eQTL) of *CALM1‐3* were used as exposures and the summary GWAS data from European individuals with MASLD were employed as outcome (Figure 9g).^[^ ^37^ ^]^ As expected, SMR analysis revealed that genetically proxied higher expression levels of *CALM1* (OR 0.95, 95% CI 0.9–1) and *CALM3* (OR 0.88, 95% CI 0.78–0.99) were causally associated with reduced risks of MASLD (Figure 9h). Taken together, these results support the crucial role of calmodulin in the development of human MASLD, highlighting its potential as a promising target in both preventing and treating this liver disease.

## 3 Discussion

In the present study, we established the therapeutic role of alnustone in MASLD and MASH by integrating the data of various mouse models. Our results provide compelling evidence that alnustone administration effectively ameliorates hepatic steatosis, inflammation, and fibrosis in both male and female mice, while also markedly improving glucose intolerance and insulin resistance. In view of the feasibility of oral medicines in clinical applications, we further proved that oral administration of alnustone also showed impressive therapeutic benefits comparable to those observed with intraperitoneal injection. Mechanistically, combining the results of mitochondrial activity assays and LiP‐SMap identified CaM as the direct target of alnustone, which acts through enhancing cytosolic Ca^2+^ levels and promoting mitochondrial fatty acid β‐oxidation (Figure 9i). Furthermore, we confirmed the essential role of CaM in mediating alnustone's hepatic effects as well as in the development of MASLD in humans. Together, these findings strongly support alnustone as a promising candidate for clinical development, offering substantial therapeutic advantages for managing MASLD and MASH.

Investigational treatments for MASLD and MASH have predominantly focused on particular aspects underlying the disease pathogenesis, including the regulation of energy balance, hepatic metabolism, and responses to lipotoxic liver injury, which often leads to inflammation and fibrosis. The drugs currently used for the treatment of NAFLD, such as insulin sensitizer pioglitazone, farnesoid X receptor agonist obeticholic acid, and the thyroid hormone receptor THRβ agonist Resmetirom, target various pathways. For instance, PPAR‐targeting drugs are shown to reduce hepatic steatosis, lobular inflammation and hepatocellular ballooning in nondiabetic MASH subjects.^[^ ^38^ ^]^ Farnesoid X receptor agonists reduce bile acid synthesis and improve hepatic steatosis and damage.^[^ ^39^ ^]^ However, the effectiveness of these drugs is often limited by adverse events and other concomitant metabolic disorders.^[^ ^38^, ^39^ ^]^ These limitations strongly suggest that alternate medications with improved efficacy and safety are critically needed.

Thyroid hormone receptor THRβ agonists, which act by increasing triacylglycerol hydrolysis and promoting lipid β‐oxidation, represent a class of treatments that are both effective and have a favorable safety profile.^[^ ^40^ ^]^ Resmetirom, a selective THRβ agonist, is the only FDA‐approved drug to treat MASLD.^[^ ^41^ ^]^ Alnustone also act on enhancing mitochondrial fatty acid β‐oxidation through calmodulin‐driven Ca^2^⁺ modulation, underscoring the potential of fatty acid β‐oxidation‐based therapies. This common mechanism of action underlies the observed therapeutic effects of both agents, particularly in terms of improving liver steatosis. In this context, alnustone, a natural small molecule compound extracted from the traditional Chinese herb *Alpinia katsumadai* Hayata,^[^ ^17^ ^]^ has shown promising results. As evidenced by our study, alnustone potently reversed hepatic steatosis and ameliorated fibrosis in multiple MASLD/MASH mice models by two routes of administration via facilitating fatty acid β‐oxidation, without any observable side effects. To be noted, alnustone's effects are superior over the clinically used FXR agonist OCA on ameliorating liver injury, steatosis, and fibrosis. Alnustone is sourced from widely available natural plants, making it both accessible and cost‐effective. Our data also delineate the beneficial effects of alnustone in the management of glucose metabolic dysfunctions, with marked improvements on glucose homeostasis in T2D mice.^[^ ^42^ ^]^ These dual benefits further make it a promising option for MASLD patients with comorbid diabetes or other metabolic disorders. Given the complex MASH pathogenesis and the lack of approved medications, these findings support that alnustone offers a promising, safe, and efficacious treatment option for MASLD/MASH.

Alnustone has been previously reported to possess antioxidant, antibacterial, and anti‐inflammatory properties.^[^ ^43^ ^]^ Chronic inflammation is a hallmark of MASH, leading to the activation of hepatic stellate cells and the subsequent development of fibrosis and cirrhosis.^[^ ^44^ ^]^ Previous study has reported alnustone's anti‐inflammatory effect,^[^ ^19^ ^]^ which is further substantiated in our MASH mice. This reduction in inflammation combined with improved fatty acid β‐oxidation can together lead to decreased liver cell injury and a slower progression to advanced MASH.^[^ ^45^, ^46^ ^]^ Oxidative stress is another key factor in the progression of MASH.^[^ ^47^ ^]^ Alnustone's antioxidant properties may contribute to fibrosis alleviation by reducing oxidative damage.^[^ ^48^ ^]^ The combination of anti‐inflammatory, antioxidant, and potential antibacterial properties in alnustone provides a multifaceted approach to treating MASLD and MASH. By addressing its multiple pathophysiological aspects, alnustone can offer comprehensive therapeutic benefits. However, the current research primarily focused on hepatocyte‐centric mechanisms, while the potential modulatory effects of alnustone and its target calmodulin on immune cells (e.g., hepatic macrophages, Kupffer cells, and monocytes) remain uncharacterized, which merits rigorous investigation in subsequent studies to comprehensively elucidate its therapeutic mechanisms.

Notably, alnustone demonstrated consistent efficacy in ameliorating hepatic steatosis, insulin resistance, and fibrosis in both male and female mice, despite female MASLD mice exhibited milder hepatic steatosis and fibrosis compared to males as reported.^[^ ^49^ ^]^ This suggests that alnustone's mechanism, targeting calmodulin to amplify mitochondrial fatty acid β‐oxidation, operates independently of sex hormone pathways, instead leveraging a conserved metabolic node to improve lipid metabolism. The compound's ability to improve glucose tolerance similarly in both sexes further support its broad applicability.^[^ ^42^ ^]^

Small molecule drugs, such as metformin, are known for their ability of binding to diverse biological targets, such as proteins, nucleic acids, and carbohydrates.^[^ ^50^ ^]^ Identifying the direct targets of such small molecule drugs has long been challenging. The LiP‐SMap technology addresses this by leveraging the binding affinity and stoichiometry between a small molecule and its target proteins.^[^ ^30^ ^]^ This method capitalizes on the attenuated protease susceptibility of the target protein when complexed with the small molecule, thereby resulting in a modified distribution pattern of intact protein and peptide products detectable by high‐resolution LC‐MS/MS and high‐throughput quantitative proteomics.^[^ ^30^, ^51^, ^52^ ^]^ By taking advantages of this approach, we identified calmodulin as a potential candidate target of alnustone. Subsequent molecular docking, SPR and MST assays validated that alnustone specifically binds to the EF‐hand/Ca^2+^‐binding site of CaM. Moreover, we confirmed that CaM is required for alnustone to reduce mitochondrial fatty acid β‐oxidation and triacylglycerol accumulation both in hepatocytes and in MASLD mice. Together, these results strongly support that CaM is a bona fide target of alnustone and is essential for alnustone's therapeutic effects on MASLD.

Calmodulin is a compact, ubiquitous adapter protein that plays a crucial role in enhancing the regulatory effects of Ca^2+^ on various proteins.^[^ ^32^ ^]^ Upon Ca^2+^ binding, calmodulin undergoes conformational changes that unlock its capacity to modulate protein activities.^[^ ^53^ ^]^ The interaction between Ca^2+^ and calmodulin is pivotal for the activation of downstream calmodulin kinase (CaMK) family members, leading to a cascade of phosphorylation events that regulates a variety of cellular processes. In our study, the treatment of alnustone on palmitic acid‐induced hepatocytes resulted in increases in both cytoplasmic and mitochondrial Ca^2+^ levels that were mediated by CaM. The increase of extracellular Ca^2+^ further suppressed cellular lipid accumulation. Considering alnustone directly binds to the Ca^2+^‐binding site of CaM and CaM knockdown blocks alnustone‐driven Ca^2+^ increases, we propose that alnustone facilitates mitochondrial Ca^2+^ influx either by inducing intracellular Ca^2+^ re‐distribution through disrupting the interaction between Ca^2+^ and CaM, or by facilitating extracellular Ca^2+^ inward flow through CaM‐related L‐type voltage‐dependent Ca^2+^ channel,^[^ ^54^ ^]^ thus leading to enhanced mitochondrial fatty acid β‐oxidation.^[^ ^55^, ^56^ ^]^ Apart from this, it has been reported that CaMKII, a downstream target of CaM, is also involved in regulating the expression and function of key regulators in fatty acid metabolism.^[^ ^57^, ^58^, ^59^ ^]^ Together, these data demonstrated a crucial role for alnustone in improving intracellular Ca^2+^ dynamics, mitochondrial fatty acid β‐oxidation, and hepatic lipid accumulation through CaM.

Interestingly, our results revealed that CaM is downregulated in livers of mouse and human patients with MASLD and MASH, supporting its critical role in the pathophysiology of this disease. Genetically proxied *CALM* expression is causally associated with the risk of human MASLD. This indicates that calmodulin may serve as a promising biomarker and therapeutic target for MASLD. Given the comprehensively beneficial roles of alnustone on MASLD mice, future clinical studies are warranted to further evaluate its therapeutic potentials in human MASLD and MASH.

In summary, combining data from multiple MASLD mouse models, hepatocellular systems, and human MASLD discoveries, the current study establishes alnustone as an effective natural compound for the treatment of MASLD/MASH by facilitating mitochondrial fatty acid β‐oxidation. We further identified calmodulin as a promising target that may have translational potentials for the treatment of human MASLD. Given these results, alnustone administration represents a promising therapeutic option for MASLD/MASH that is both effective and safe.

## 4 Experimental Section

### MASLD Mice Models and Alnustone Interventions

All animal studies were approved by the Institutional Review Board of the Center for Reproductive Medicine, Shandong University (Approval Number: IRB 2022–56). The animals received humane care according to the Guide for the Care and Use of Laboratory Animals published by the National Academy of Sciences and the National Institutes of Health. Wildtype C57BL/6J (8‐week‐old) male and female mice were purchased from GemPharmatech Co. (Jiangsu, China). Mice were allowed to acclimatize for 1 weeks and housed under specific pathogen‐free conditions in a temperature‐controlled room (22‐25 °C) with a 12‐h light/dark cycle and free access to food and water. All mice were visually inspected every week during cage change. The sample size, sex, and age of the animals used were specified in the text and/or figure legends. All the in vivo experiments were run using a double‐blind procedure.

To establish a MASLD model, C57BL/6J mice were fed a high‐fat diet (60% kcal fat, D12492, Research Diets, New Brunswick, USA) beginning at the age of 8 weeks for 12 weeks. Alnustone was purchased from TargetMol (T4S0176, CAS 33457‐62‐4), dissolved in DMSO, and stored at ‐20 °C for at most a week. After MASLD established, the mice were randomly grouped, either receiving vehicle (5% DMSO in corn oil) by intraperitoneal injection, 10 mg kg^−1^ alnustone (dissolved in 5% DMSO in corn oil) by intraperitoneal injection, vehicle (5% DMSO and 0.5% CMC‐Na) by gavage, or 30 mg/kg alnustone (dissolved in 5% DMSO and 0.5% CMC‐Na) by gavage every day. The intraperitoneal dosage of alnustone was chosen based on previously published studies and the oral dosage was adjusted based on a dose‐equivalent conversion from the intraperitoneal injection dose, following standard pharmacokinetic principles.^[^ ^42^, ^60^, ^61^ ^]^ Both male and female mice were used for experiments as above. After 1 week, we performed GTT and ITT to assess the effect on glucose metabolism of alnustone. After 2 weeks, the mice were sacrificed, and their livers were analyzed.

To test the therapeutic effect of diabetic MASLD model, the *db*/*db* mice were purchased from Vital River Laboratory Animal Technology Co (Beijing, China) and were fed with standard chow diet (Beijing KEAO XIElI FEED Co., China). Alnustone (10 mg kg^−1^) or vehicle was administered for 5 days by intraperitoneal injection.

### MASH Mice Models and Alnustone Interventions

To investigate the therapeutic potential of alnustone in MASH, two independent animal models were established. 8‐week‐old male C57BL/6J mice were fed with a MCD diet (A02082002BR, Research Diets, New Brunswick, USA) for 4 weeks. After MASH established, mice were randomly divided into groups and treated with alnustone (10 mg kg^−1^ day^−1^) by intraperitoneal injection or 30 mg kg^−1^ obeticholic acid (OCA; Selleckchem, Houston, TX) by gavage every day for 2 weeks. The dosing scheme was also shown in figures and illustrated in the Results section.

In another model, 8‐week‐old male C57BL/6J mice were subjected to a 22‐week AMLN diet (40% kcal fat, 2% kcal cholesterol, 22% kcal fructose. After MASH was established, mice were randomly divided into groups and treated with alnustone (10 mg kg^−1^ day^−1^) by intraperitoneal injection. The AMLN diet feeding continued until the experimental endpoint to mimic chronic disease exposure during therapeutic intervention. All treatments were maintained for 14 consecutive days, with the experimental scheme shown in figures and illustrated in the Results section.

### Construction of Hepatic CaM Knockdown Mice

For the generation of *Calm1/2/3* knockdown mice, Ad‐sh‐GFP and Ad‐sh‐*Calm1‐3* adenoviruses were constructed (Obio Technology, Shanghai, China). The adenoviral vector (pADV‐U6‐shRNA‐CMV‐EGFP) was designed with shRNA sequences targeting *Calm1*, *Calm2*, and *Calm3* under the U6 promoter, with CMV‐driven EGFP allowed for tracking transduction efficiency. The shRNA sequences are as follows: *Calm1*: CGACGGACAAGUCAACUAUTT; *Calm2*: CGGAUGGUAAUGGCACAAUTT; *Calm3*: GAGAUGGCCAGGUCAAUUATT. After HFD feeding for 12 weeks, adenoviruses were delivered by tail vein injection (3 × 10^9^ PFU per mouse) to male C57BL/6J mice for 14 days to construct CaM knockdown mice.

### Human Samples

Liver tissues from control subjects and MASLD patients were collected from cases with partial hepatectomy performed in the Department of Hepatobiliary Surgery in Shandong Provincial Hospital as previously reported.^[^ ^62^ ^]^ MASLD human samples strictly adhered to the disease diagnostic criteria,^[^ ^63^ ^]^ including histological confirmation of steatosis (>5%) and at least one cardiometabolic risk factor. Participants with excessive alcohol consumption (alcohol intake >20 g day^−1^ for females and >30 g day^−1^ for males) were excluded. Clinical information detailed in Table S2 (Supporting Information). Human studies were conducted according to the guidelines of the Declaration of Helsinki and approved by Ethic Committee of Shandong Provincial Hospital (Approval Number: SWYX‐2022468). Informed consent was obtained from each individual and individuals’ clinical biochemical data were extracted from medical records.

### Cell Culture and Treatment

The HepG2 and AML12 cell lines were purchased from the Type Culture Collection of the Chinese Academy of Sciences (Shanghai, China). HepG2 cell lines were grown in high‐glucose DMEM medium (Gibco, USA) supplemented with 10% fetal bovine serum (FBS) (Gibco, USA) and 1% penicillin‐streptomycin. AML12 cell line was cultured in DMEM/ F12 medium (Gibco, USA) containing 10% FBS, 1% ITS liquid media supplement (Sigma, USA), 40 ng mL^−1^ dexamethasone, and 1% penicillin‐streptomycin. All cell lines were kept in a humidified incubator at 37 °C and 5% CO_2_. Cells were used from third to tenth passage in each experiment. The cell lines were assessed for mycoplasma contamination, and the test results were negative.

To establish a cell model of lipid accumulation, 0.2 mM palmitic acid (dissolved in 0.25% fatty acid–free BSA) was added into medium. Fatty acid‐free BSA (0.25%) alone was used as a vehicle control. Meanwhile, alnustone dissolved in DMSO was added at a dose of 5, 10, 20 µM and DMSO was added as a control.

For extracellular calcium treatment, calcium chloride (CaCl_2_) was purchased from Sigma (St. Louis, MO, USA) and added into medium of AML12 cells at a final dose of 1.5, 2, or 2.5 mM. DMEM/ F12 medium including 1 mM calcium was used as a control.

For cellular Oil Red O staining, cells were cultured in six‐well plates and after palmitic acid and alnustone treatment, culture medium was removed, and cells were fixed with 4% paraformaldehyde for 15 min and washed with PBS three times. Then the cells were treated with 60% isopropanol for 5 min, removed isopropanol, stained with Oil Red O working solution (Sigma, USA) for 15 min, and then washed with dH_2_O. Then the cells were stained with hematoxylin for 2 min and washed with dH_2_O at least five times. After Oil Red O staining were observed and took photos using an inverted phase contrast microscope (Olympus, Japan), the cells were lysed by 4% NP‐40 (dissolved in isopropanol). The released oil red O‐stained lipids were quantified using a spectrophotometer at the absorbance 490 nm. For analysis of ATP in cells, hepatocytes incubated with DMSO or alnustone (10 µM) for 24 h were measured by Enhanced ATP Assay Kit (Beyotime) according to the manufacturer's protocols.

### Knockdown of CaM by Small Interfering RNA

AML12 cells were transfected with small interfering RNA (siRNA) for knockdown of CaM in vitro. Scramble siRNA was used as the control. The sequences of siRNA (5′ to 3′) were as follows: *Calm1*: sense CGACGGACAAGUCAACUAUTT, antisense AUAGUUGACUUGUCCGUCGTT; *Calm2*: sense CGGAUGGUAAUGGCACAAUTT, antisense AUUGUGCCAUUACCAUCCGTT; *Calm3*: sense GAGAUGGCCAGGUCAAUUATT, antisense UAAUUGACCUGGCCAUCUCTT.

### Primary Hepatocyte Isolation

Primary hepatocytes were isolated from 8‐week‐old male C57BL/6J mice using a two‐step collagenase perfusion procedure. Briefly, mouse livers were perfused with calcium‐free HBSS (Life Technologies) followed by perfusion with type IV collagenase (Gibco) through the portal vein. Hepatocytes were flushed out and filtered through a 70 µm cell strainer. Cells were washed for three times and centrifuged for 10 min at 50 g to reduce the amount of nonparenchymal cells at 4 °C. Cell viability was determined with trypan blue dye. Plates with cell viability greater than 80% were used for experiments. Isolated primary hepatocytes were seeded in six‐well plates at 1 × 10^5^ per well and cultured in DMEM medium containing 10% Fetal bovine serum and 1% penicillin‐streptomycin in a 5% CO_2_ incubator at 37 °C.

### Triacylglycerol and Total Cholesterol Assays

Serum was collected by centrifugation at 3000 rpm for 15 min at 4 °C. Fasting serum triacylglycerol and total cholesterol levels were determined enzymatically according to the manufacturer's guidance (Applygen, Beijing, China).

Liver triacylglycerol content was measured from frozen tissue lysates. Frozen liver samples were weighed prior to homogenization in PBS. Lysates were centrifuged at 12 000 g for 10 min at 4 °C before the supernatant was extracted. Triacylglycerol and total cholesterol levels were determined enzymatically according to the manufacturer's guidance (Applygen, Beijing, China). Each sample was then normalized to the protein content.

### Histology Analysis

For H&E, Masson, Sirius red and immunohistochemistry staining, fresh liver tissues were immersion‐fixed in 4% paraformaldehyde for at least 24 h and stored at 4 °C. Then paraffin‐embedded tissue was cut into 5 µm sections. H&E staining was performed to visualize lipid accumulation. Liver fibrosis was evaluated by Masson staining and Sirius red staining. Staining was conducted following the standard protocols. For immunohistochemistry, antigen retrieval was performed by heating in Tris/EDTA buffer pH 9.0 (Invitrogen). After cooling to room temperature, sections were washed with PBS and incubated with hydrogen peroxide to block endogenous peroxidase activity. Liver sections were incubated overnight at 4 °C with primary anti‐Calmodulin 1/2/3 at 1:500 (abcam, ab45689), and then incubated with HRP‐conjunct anti‐rabbit/mouse secondary antibody (ZSGB‐BIO, PV‐9000). All sections were counterstained with DAB (Vector, SK‐4100) and hematoxylin. Cell apoptosis was measured using the One Step TUNEL Apoptosis Assay Kit (Beyotime) following the manufacturer's instructions. The percentage of Tunel cells was calculated and at least 3000 cells per animal were counted.

For Oil Red O staining, frozen liver tissues were embedded with optimum cutting temperature compound and sectioned at 8 µm. One to two representative images were taken per section with a light microscope at 40X magnification (Leica, Germany). Images were assessed and quantitated by Image J software.

### RNA Isolation and Real‐Time Quantitative PCR

We extracted total RNA from isolated frozen live, heart, kidney, white adipose tissue, brown adipose tissue, and muscle using Trizol reagent (Invitrogen, USA) and followed the manufacturer's protocols. Isolated RNAs were reverse transcribed using the Prime Script RT Kit with gDNA Eraser (Takara, Shiga, Japan). Real‐time quantitative PCR was performed using SYBR Premix Ex Taq Kit (Takara, Shiga, Japan) on a Light Cycler 480 System (Roche, Basel, Switzerland). Results were normalized to *β‐Actin* mRNA levels. The primers used in the current article were listed in Table S3 (Supporting Information).

### GTT and ITT

For glucose tolerance test (GTT), mice were fasted overnight for 16 h and measured fasting blood glucose on tail vein using OneTouch Ultra glucometers (LifeScan). Then the mice were injected with 20% glucose (2 g kg^−1^ body weight) intraperitoneally and blood glucose was recorded at 15, 30, 60, and 120 min. Area under the curve was calculated as an index of whole glucose excursion in the blood after glucose loading.

For insulin tolerance test (ITT), mice were fasted for 6 h starting at 8 am and measured fasting blood glucose. Then the mice were injected with insulin at a dose of 0.75 IU kg^−1^ body weight and blood glucose was recorded at 15, 30, 60, 120 min.

### Liver Hydroxyproline Content

Liver samples were weighed and hydrolyzed with an alkaline hydrolysis solutionwere to degrade collagen. Samples were centrifuged and hydroxyproline content was measured using a colorimetric assay (Nanjing Jiancheng Bioengineering Institute), according to the manufacturer's instructions.

### Flow Cytometry Assay

To investigate the hepatic immune cell infiltration in mice fed with AMLN diet in combination with administrated with vehicle/alnustone, the mice were anesthetized with isoflurane and underwent liver perfusion. The liver was then mechanically dissociated and digested at 37 °C for 30 min in RPMI 1640 medium (C11875500BT, Gibco, USA) supplemented with 0.01% collagenase IV (17 104 019, Gibco, USA), 0.02% BSA (V900933, Sigma‐Aldrich, USA), and 0.001% DNase I (DN25, Sigma‐Aldrich, USA). The single‐cell suspension was purified by 40% Percoll (P8370, Solarbio, China) density gradient centrifugation and analyzed using flow cytometry (BD Biosciences). To exclude dead cells from the analysis, the Zombie NIR™ Fixable Viability Kit (423 106, BioLegend, CA, USA) was utilized. For cell surface staining, hepatic immune cells were subjected to incubation with various antibodies in a staining buffer (comprising PBS and 2% FBS) for 30 min at 4 °C in darkness. The antibodies used were as follows: CD3‐FITC (clone 17A2), CD19‐BV510 (clone 6D5), CD11b‐BV421 (clone M1/70), F4/80‐PE/Cyanine7 (clone BM8), Ly6G‐BV785, Ly6C‐APC (clone HK1.4) these antibodies were purchased from BioLegend, USA. CD45‐Alexa Fluor™ 700 (clone 30‐F11) was purchased from Invitrogen, USA. Flow cytometry data were analyzed with FlowJo software v10.8.1 (TreeStar).

### Lipid Extraction

Lipids were extracted from approximately 30 mg of frozen tissues using a modified version of the Bligh and Dyer's method. Briefly, tissues were homogenized in 900 µL of chloroform: methanol: MilliQ H_2_O (3:6:1) (v/v/v). The homogenate was then incubated at 1500 rpm for 30 min at 4 °C. At the end of the incubation, 350 µL of deionized water and 300 µL of chloroform were added to induce phase separation. The samples were then centrifuged and the lower organic phase containing lipids was extracted into a clean tube. Lipid extraction was repeated once by adding 500 µL of chloroform to the remaining aqueous phase, and the lipid extracts were pooled into a single tube and dried in the SpeedVac under OH mode. Samples were stored at ‐80 °C until further analysis.

### Lipidomics Analyses

Lipidomic analyses were conducted using a Jasper HPLC coupled with Sciex TRIPLE QUAD 4500 MD as reported previously.^[^ ^64^ ^]^ Separation of individual lipid classes by normal phase (NP)‐HPLC was carried out using a TUP‐HB silica column (i.d. 150×2.1 mm, 3 µm) with the following conditions: mobile phase A (chloroform: methanol: ammonium hydroxide, 89.5:10:0.5) and mobile phase B (chloroform: methanol: ammonium hydroxide: water, 55:39:0.5:5.5). MRM transitions were set up for comparative analysis of various lipids. Individual lipid species were quantified by referencing to spiked internal standards. d9‐PC32:0(16:0/16:0), PE 34:0, dic8‐PI, d31‐PS, C17:0‐PA, DMPG, CL‐14:0, C14‐BMP, C12‐SL, C17‐LPC, C17‐LPE, C17:1‐LPI, C17:0‐LPA, C17:1‐LPS, C17‐Cer, C12‐SM, d17:1‐S1P, d17:1‐Sph, C8‐GluCer, C8‐LacCer, Gb3‐d18:1/17:0, d3‐16:0 carnitine, DAG(18:1/18:1)‐d5 were obtained from Avanti Polar Lipids. GM3‐d18:1/18:0‐d3 was purchased from Matreya LLC. Free fatty acids were quantitated using d31‐16:0 (Sigma‐Aldrich). d6‐CE 18:0 and TAG (16:0)3‐d5 were obtained from CDN isotopes.

### Seahorse Analysis

To assess mitochondrial respiratory functions, oxygen consumption rates were analyzed using a XF96 Analyzer (Agilent Technologies, CA, USA). Briefly, AML12 cells were plated at 7000 cells per well in XF 96‐well microplates and cultured at 37 °C with 5% CO_2_. The adherent cells were then challenged by 0.2 mM palmitic acid and treated with vehicle or alnustone (10 µM) for 24 h. Cells were incubated in pre‐warmed assay medium (XF base medium with 2 mM glucose, 0.5 mM carnitine, 0.2 mM PA/BSA) with or without alnustone for 1 h without CO_2_ before oxygen consumption analysis. The baseline oxygen consumption rate (OCR) was recorded, and then continuous injections through ports in the XF Assay cartridges of pharmacologic inhibitors: oligomycin (1.5 µM), carbonyl cyanide4‐(trifluoromethoxy) phenylhydrazone (FCCP 1 µM), rotenone and antimycin A (0.5 µM) sequentially. The injections were performed during continuous oxygen measurements according to the manufacturer's instructions. The OCR was adjusted by protein content.

### LiP‐SMap Analysis

Lip‐SMap and data analysis were performed according to the standard protocols. Briefly, hepatocytes were lysed in phosphate buffered saline (PBS). The supernatant was retained and protein concentrations were measured with a BCA assay kit (Beyotime). Then the lysates were added equally to six tubes and incubated with 10 µM alnustone or vehicle DMSO (three replicates per group). Limited proteolysis was employed by incubation with protease K and trypsin, followed with mass spectrometry. To further determine the regulatory network of alnustone, protein‐protein interaction analysis of potential binding protein was conducted using STRING database.

### Molecular Docking

The X‐ray crystal structures of Calmodulin (PDB: 1UP5) were retrieved from the Protein Data Bank. The protonation state of all the compounds was set at pH = 7.4, and the compounds were expanded to 3D structures using Open Babel. AutoDock Tools (ADT3) were applied to prepare and parametrize the receptor protein and ligands. The docking grid documents were generated by AutoGrid of sitemap, and AutoDock Vina (1.2.0) was used for docking simulation. The optimal pose was selected to analysis interaction. Finally, the protein‐ligand interaction figure was generated by PyMOL.

### SPR Assay

The interaction between the recombinant calmodulin and alnustone was quantified using a Biacore 1K (Cytiva, Sweden) with a CM5 sensor chip. The 300 mmol L^−1^ concentration of buffer salts and 0.1% BSA were used to reduce the non‐specific binding on the chip. The amine coupling method was applied to immobilize recombinant calmodulin on the Sencor chip. Calmodulin coupling was performed at a concentration of ≈30–50 µg mL^−1^ in a pH 4.0 sodium acetate solution, with a chip activation time of 60 s and a closure time of 180 s. Small molecules were solubilized with 5% DMSO, and PBST was used as the running buffer. Biacore Insight Evaluation (5.0.18.22102) was used to calculate the kinetic parameters of Kd.

### MST Assay

Recombinant calmodulin was labeled with a Monolith Protein Labeling Kit (red‐NHS 2nd Generation Kit), followed by the addition of different concentrations of alnustone (0.122 nM to 1 uM). After incubation for 15 min, Microscale thermophoresis (MST) assay was performed at room temperature on a Monolith NT.115 instrument (Nano Temper) by the manufacturer's protocol. Data analysis was performed with Nano Temper Analysis software.

### Cytosolic Ca^2+^ and Mitochondrial Ca^2+^ Measurement

Cytosolic and mitochondrial Ca^2+^ level in AML12 cell line was determined using the Ca^2+^ indicator Fluo‐4 AM and Rhod‐2 AM (Sigma), respectively. Cells were seeded on poly‐L‐lysine‐coated glass bottom culture dish were then challenged by 0.2 mM palmitic acid and treated with vehicle or alnustone (10 µM) for 24 h. Then cells were washed with PBS three times and loaded with 2 µM Fluo‐4 AM or Rhod‐2 AM combined with Pluronic F‐127 (Invitrogen, CA, USA) at 37 °C for 30 min. Then cells were washed with indicator‐free buffer and incubated for a further 30 minutes.

After rinsing, images were captured by the confocal laser scanning microscope (Andor Technology PLC, Andor Dragonfly 200). Measurements of fluorescence intensities were performed by ImageJ software (National Institutes of Health). Briefly, all images were first converted to 8 bits, and immunostaining was segmented by manual thresholding with constant parameters. The fluorescence intensity of the segmented signal was then quantified using the measurement tool.

### Summary‐Data‐Based Mendelian Randomization

Summary‐data‐based Mendelian randomization (SMR) was employed to estimate the causal associations of *CALM1*, *CALM2*, or *CALM3* expression with the risk of MASLD. Summary GWAS data of 32941 European ancestry individuals with non‐alcoholic fatty liver disease were used in this study.^[^ ^37^ ^]^ The dataset of blood expression quantitative trait loci (eQTL) data was extracted from the eQTLGen consortium which included 31 684 individuals and selected by passing a *P*‐value threshold of 5.0 × 10^−8^. Heterogeneity in the dependent instrument (HEIDI) test was applied to distinguish pleiotropy from linkage, where P‐HEIDI <0.01 were considered likely due to pleiotropy and thus discarded from the analysis. SMR and HEIDI tests were implemented using the SMR software tool (SMR v1.3.1). To account for multiple testing, the *P*‐values were adjusted to control the false discovery rate (FDR).

### Statistical Analysis

The results were expressed as mean ± SEM for the indicated number of observations. A two‐tailed unpaired Students *t* test or rank sum test was performed to compare two groups. Comparisons among multiple groups were determined by one‐way ANOVA followed by Tukey's multiple comparison test using Prism 8 (Graphpad Software, San Diego, CA). Linear regression analysis was used to determine the correlation between gene expression level and clinical parameters using SPSS v27.0 software (SPSS Inc., Chicago, IL, USA). For human studies, the clinical sample size was statistically powered (>80% at α = 0.05) based on preliminary effect sizes, and significance is assessed by two‐tailed unpaired Student's *t* test. Differences with *P*<0.05 were considered statistically significant.

## Conflict of Interest

The authors declare no conflict of interest.

## Supporting information



Supporting Information

## Data Availability

The data that support the findings of this study are available from the corresponding author upon reasonable request.
