# Supplementary material for: Alnustone Ameliorates Metabolic Dysfunction‐Associated Steatotic Liver Disease by Facilitating Mitochondrial Fatty Acid β‐Oxidation via Targeting Calmodulin
Source: Adv Sci (Weinh). 2025 Jun 5;12(31):e11984. doi: 10.1002/advs.202411984 (PMC12376512; doi:10.1002/advs.202411984)
Supplement: Supplementary file 1 — Supporting Information [file ADVS-12-e11984-s001.pdf]

ADVANCED SCIENCE
Open Access

Supporting Information

for *Adv. Sci.*, DOI 10.1002/advs.202411984

Alnustone Ameliorates Metabolic Dysfunction-Associated Steatotic Liver Disease by Facilitating Mitochondrial Fatty Acid β-Oxidation via Targeting Calmodulin

Shourui Hu, Xiaofan Liang, Yiming Qin, Yuxuan Li, Yue Liu, Congcong Liu, Zongxuan Lin, Chunxuan Geng, Yanqi Xu, Daimin Wei, Yingying Qin, Han Zhao, Yuqing Zhang* and Zi-Jiang Chen*

Supporting Information

Alnustone Ameliorates Metabolic Dysfunction-associated Steatotic Liver Disease by Facilitating Mitochondrial Fatty Acid β-oxidation via Targeting Calmodulin

Shourui Hu, Xiaofan Liang, Yiming Qin, Yuxuan Li, Yue Liu, Congcong Liu, Zongxuan Lin, Chunxuan Geng, Yanqi Xu, Daimin Wei, Yingying Qin, Han Zhao, Yuqing Zhang,* and Zi-Jiang Chen*

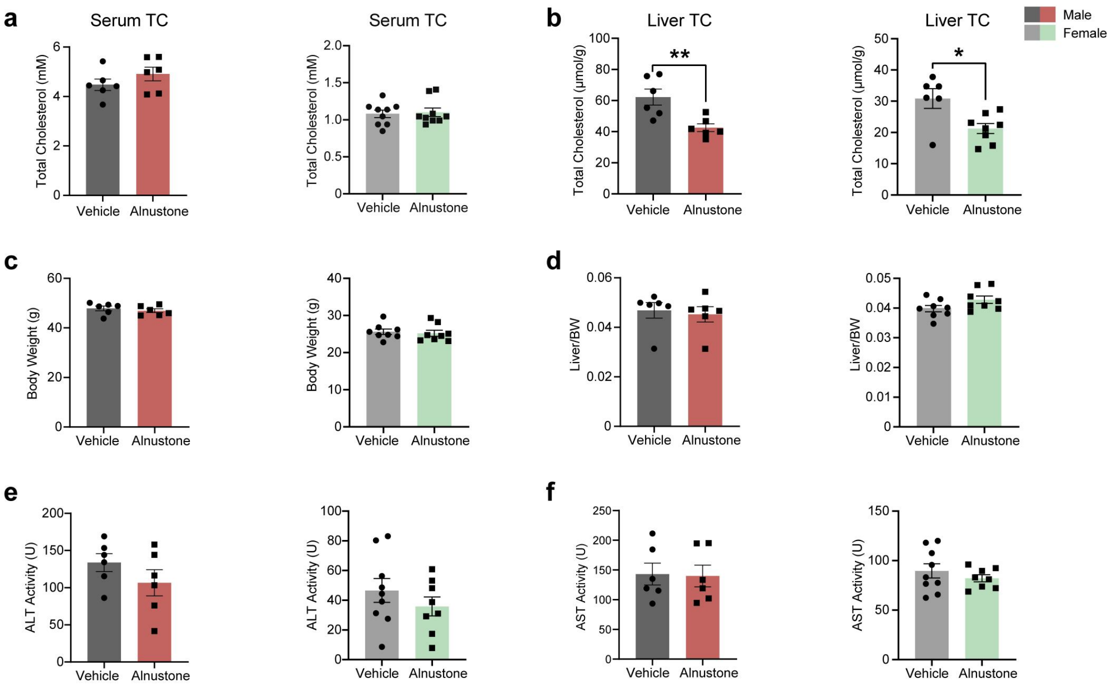

Figure S1. Alnustone reduces hepatic total cholesterol levels and does not worsen liver function in MASLD mice.

(**a**) Serum total cholesterol levels of male (n=6) and female (n=9) mice fed with HFD and administrated with vehicle/alnustone for 2 weeks.

(**b**) Hepatic total cholesterol levels of male (n=6) and female (vehicle: n=6, alnustone: n=8) mice fed with HFD and administrated with vehicle/alnustone for 2 weeks. Total cholesterol contents were normalized by hepatic protein levels.

(**c and d**) Body weight (**c**) and liver-to-body weight ratio (**d**) of male (n=6) and female (n=8) mice fed with HFD and administrated with vehicle/alnustone for 2 weeks.

(**e and f**) Plasma levels of ALT (**e**) and AST (**f**) of male (n=6) and female (vehicle: n=9, alnustone: n=8) mice fed with HFD and administrated with vehicle/alnustone for 2 weeks.

Data are presented as mean ± SEM. **P* <0.05, ***P* <0.01; significance is assessed by two-tailed unpaired Student's *t* test.

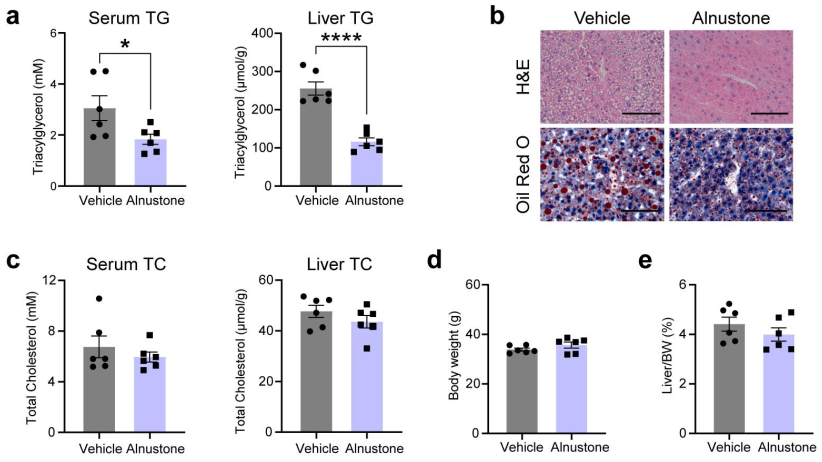

Figure S2. Alnustone alleviates hepatic steatosis in leptin receptor-deficient (*db*/*db*) mice.

(**a**) Serum and hepatic triacylglycerol levels of *db*/*db* mice (n=6) administrated with vehicle/alnustone for 5 days. Triacylglycerol contents were normalized by hepatic protein levels.

(**b**) H&E and Oil Red O staining were performed in liver sections from vehicle/alnustone-treated *db*/*db* mice. Scale bar: 50 μm.

(**c**) Serum and hepatic total cholesterol levels of *db*/*db* mice (n=6) administrated with vehicle/alnustone for 5 days. Total cholesterol contents were normalized by hepatic protein levels.

(**d and e**) Body weight (**d**) and liver-to-body weight ratio (**e**) of *db*/*db* mice (n=6) administrated with vehicle/alnustone for 5 days.

Data are presented as mean ± SEM. **P* <0.05, *****P* <0.0001; significance is assessed by two-tailed unpaired Student's *t* test.

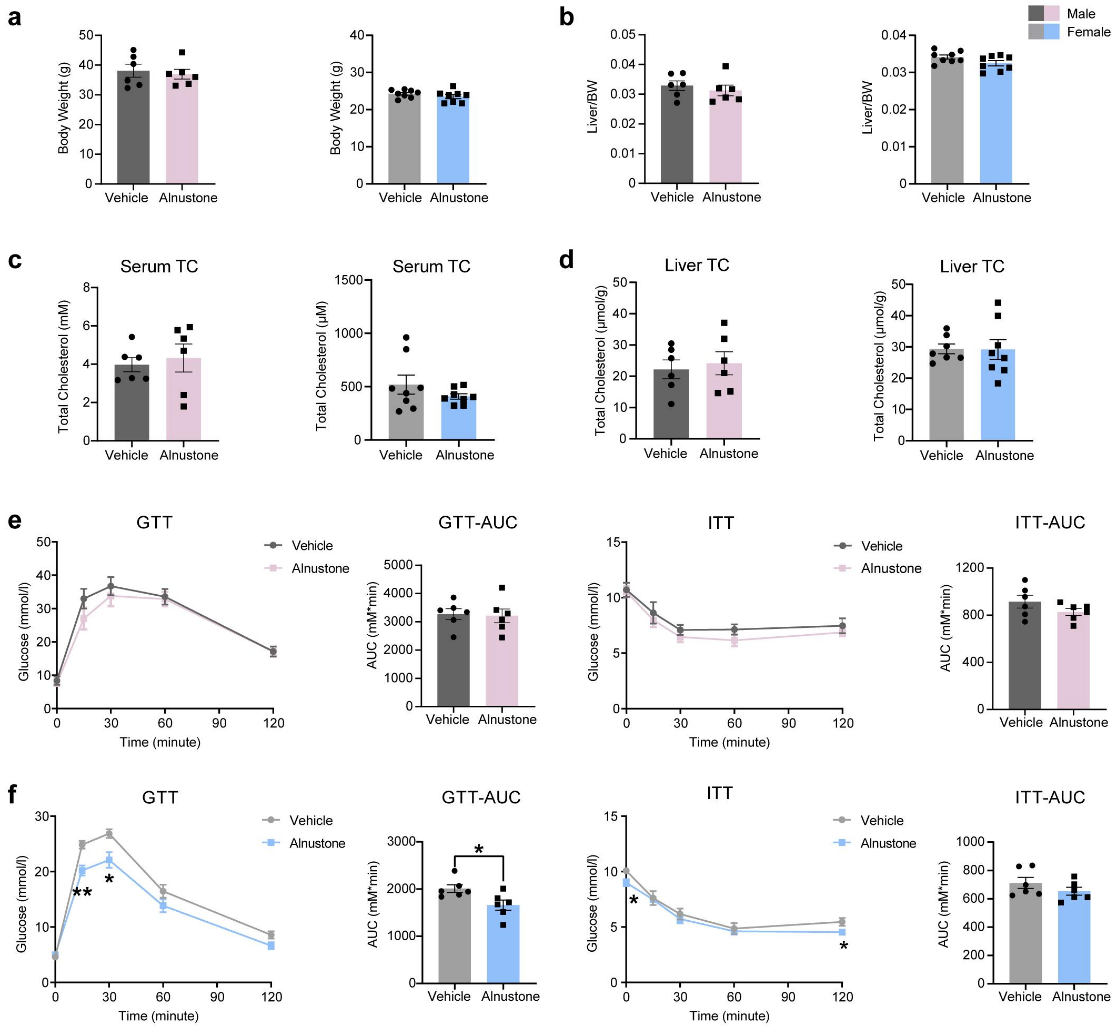

Figure S3. The effects of oral administration of alnustone on lipid metabolism and insulin resistance in male and female MASLD mice induced by high-fat diet.

(**a and b**) Body weight (**a**) and liver-to-body weight ratio (**b**) of male (n=6) and female (n=8) mice fed with HFD and administrated with vehicle/alnustone for 2 weeks.

(**c**) Serum total cholesterol levels of male (n=6) and female (n=8) mice fed with HFD and administrated with vehicle/alnustone for 2 weeks.

(**d**) Hepatic total cholesterol levels of male (n=6) and female (vehicle: n=7, alnustone: n=8) mice fed with HFD and administrated with vehicle/alnustone for 2 weeks. Total cholesterol contents were normalized by hepatic protein levels.

(**e and f**) Glucose tolerance test and insulin tolerance test were performed on male and female mice administrated with alnustone or vehicle for 1 weeks and area under curve (AUC) was calculated and compared. (n=6)

Data are presented as mean ± SEM. $^{*}P<0.05$, $^{**}P<0.01$; significance is assessed by two-tailed unpaired Student's *t* test.

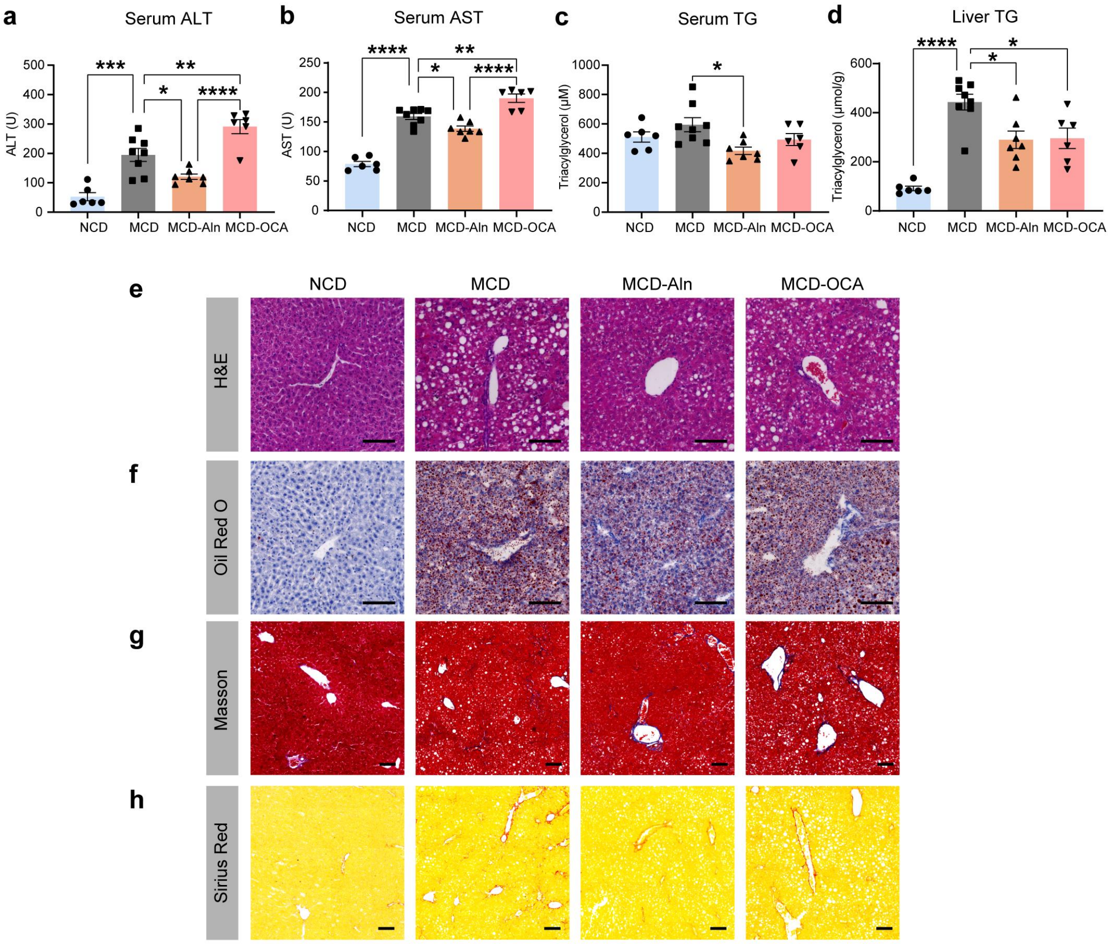

Figure S4. Superior efficacy of alnustone on MASH phenotypes in mice fed with MCD, compared with OCA treatment.

(**a-b**) Plasma levels of ALT (**a**) and AST (**b**) of mice fed with chow or MCD diets and administrated with vehicle/alnustone/OCA for 2 weeks. (NCD: n=6, MCD: n=8, MCD-aln: n=7, MCD-OCA: n=6)

(**c**) Serum triacylglycerol levels of mice fed with chow or MCD diets and administrated with vehicle/alnustone/OCA for 2 weeks. (NCD: n=6, MCD: n=8, MCD-aln: n=7, MCD-OCA: n=6)

(**d**) Hepatic triacylglycerol levels of mice fed with chow or MCD diets and administrated with vehicle/alnustone/OCA for 2 weeks. Triacylglycerol contents were normalized by hepatic protein levels. (NCD: n=6, MCD: n=8, MCD-aln: n=7,

MCD-OCA: n=6)

(**e-h**) H&E (**e**), Oil Red O (**f**), Masson (**g**), and Sirius red (**h**) staining were performed in liver sections from vehicle/alnustone/OCA-treated MCD mice. Scale bar: 100 μm. Data are presented as mean ± SEM. **P* <0.05, ***P* <0.01, ****P* <0.001, *****P* <0.0001; two- tailed unpaired Student's *t* test for Chow vs. Vehicle and One-way ANOVA, with Tukey's multiple comparisons post-hoc test, chow controls excluded.

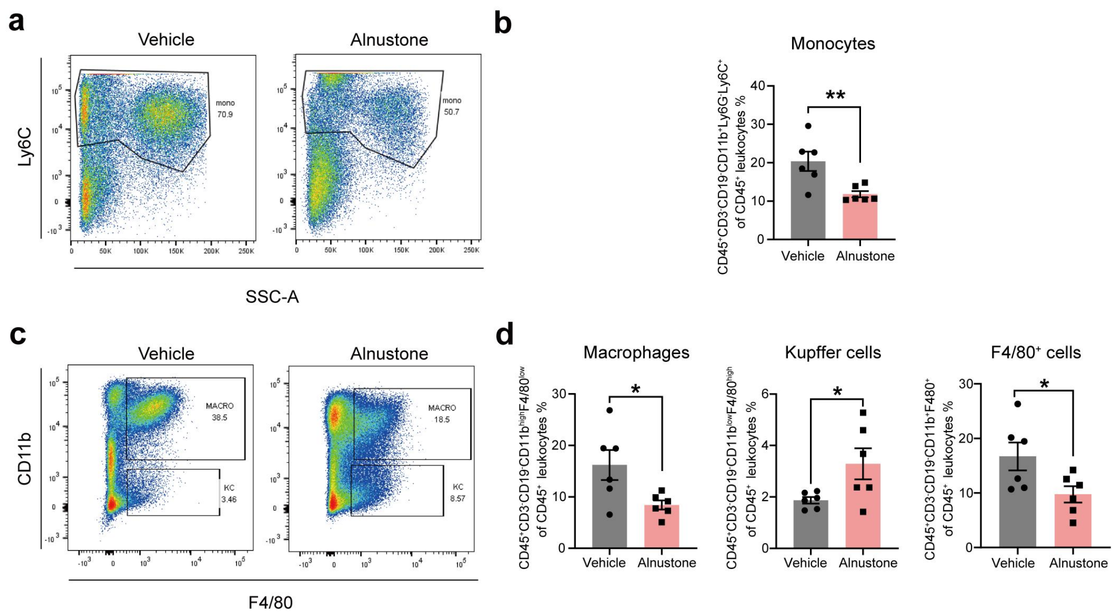

Figure S5. Alnustone attenuates hepatic inflammation in MASH mice model.

(**a**) Flow cytometry plots of hepatic monocytes ($CD45^{+}CD3^{-}CD19^{-}CD11b^{+}Ly6G^{-}Ly6c^{+}$) in the livers of AMLN-fed mice and administrated with vehicle/alnustone for 2 weeks.

(**b**) The percentage of monocytes in the livers of AMLN-fed mice and administrated with vehicle/alnustone for 2 weeks.

(**c**) Flow cytometry plots of macrophages ($CD45^{+}CD3^{-}CD19^{-}CD11b^{high}F4/80^{low}$) and KCs ($CD45^{+}CD3^{-}CD19^{-}CD11b^{low}F4/80^{high}$) in the livers of AMLN-fed mice and administrated with vehicle/alnustone for 2 weeks.

(**d**) The percentage of macrophages, KCs and F4/80 positive cells in the livers of AMLN-fed mice and administrated with vehicle/alnustone for 2 weeks.

Data are presented as mean ± SEM. *P <0.05, **P <0.01; significance is assessed by two-tailed unpaired Student's t test.

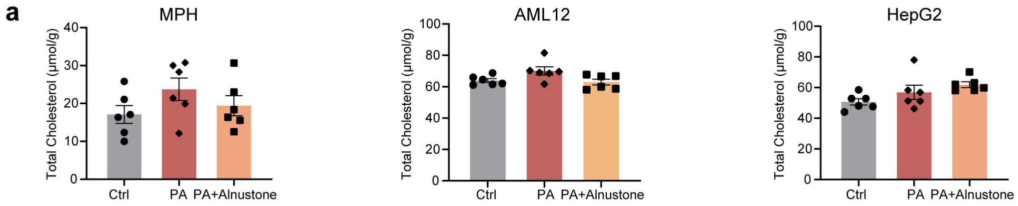

Figure S6. Hepatocytes treated with or without alnustone exhibit similar cholesterol accumulation.

(**a**) Analysis of cellular total cholesterol levels in mouse primary hepatocytes, AML12, and HepG2 cells treated with 0.2 mM palmitic acid and 10 μM alnustone for 24h. (n=6)

Data are presented as mean ± SEM.

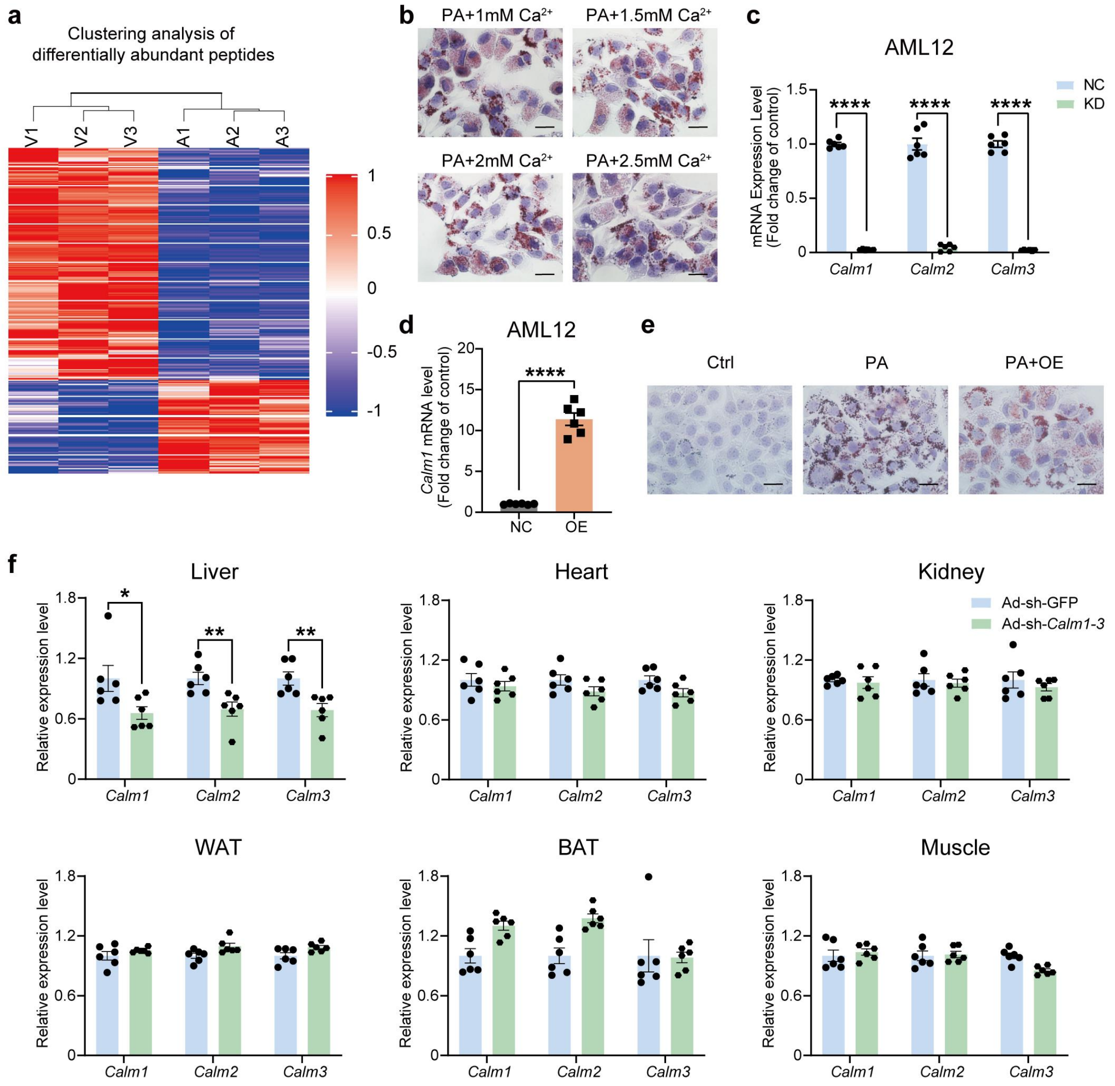

Figure S7. Calmodulin knockdown blunts alnustone's effect on lipid metabolism.

(a) Clustering analysis of differentially abundant peptides identified by LiP-SMap.

(b) AML12 cells treated with 1μM, 1.5μM, 2μM and 2.5μM $CaCl_2$ were stained with Oil Red O, pictured using an inverted phase contrast microscope. Scale bar: 20 μm.

(c) Efficiency of *Calm1*, *Calm2* and *Calm3* knockdown (KD) in AML12 cells. (n=6)

(d) Efficiency of *Calm1* overexpression (OE) in AML12 cells. (n=6)

(e) AML12 cells with or without *Calm1* overexpression in the presence of palmitic acid were stained with Oil Red O, pictured using an inverted phase contrast microscope. Scale bar: 20 μm.

(f) Male C57BL/6J mice with HFD diets were injected with Ad-sh-GFP or Ad-sh-*Calm1-3* and administrated with vehicle/alnustone for 2 weeks. Quantification of *Calm1*, *Calm2* and *Calm3* relative to *β-Actin* in liver, heart, kidney, white adipose tissue (WAT), brown adipose tissue (BAT), and muscle from mice treated with Ad-sh-GFP or Ad-sh-*Calm1-3*. (n=6)

Data are presented as mean ± SEM. **P* <0.05, ***P* <0.01, *****P* <0.0001; significance is assessed by two-tailed unpaired Student's *t* test.

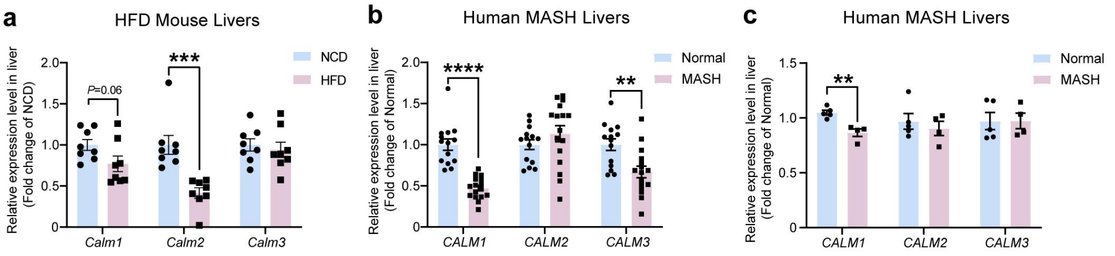

Figure S8. Calmodulin expression is decreased in MASLD mice induced by high-fat diet and MASH patients.

(**a**) Gene expression levels of *Calm1/2/3* mRNA in the livers of mice fed a NCD or HFD from the GEO GSE109327 dataset.

(**b**) Gene expression levels of *CALM1/2/3* mRNA in the livers of normal control subjects and MASH patients from the GEO GSE126848 dataset.

(**c**) Gene expression levels of *CALM1/2/3* mRNA in the livers of normal control subjects and MASH patients from the GEO GSE147304 dataset.

Data are presented as mean ± SEM. $^{**}P$ <0.01, $^{***}P$ <0.001, $^{****}P$ <0.0001; significance is assessed by two-tailed unpaired Student's *t* test.

Table S1. The metabolic phenotypes of MCD mice received treatments, corresponding to animals in Figure S4.

Phenotype	NCD	MCD-Vehicle	MCD-Alnustone	MCD-OCA
ALT (U)	52.57 ± 13.8	195 ± 22.2<br>P=0.0003a	120.7 ± 9.01<br>P=0.032*; P<0.0001#	291.3 ± 23.97
AST (U)	78.83 ± 4.43	159.4 ± 5<br>P<0.0001a	138.7 ± 4.5<br>P=0.033*; P<0.0001#	190.2 ± 7.18
Serum TG (μM)	511.4 ± 34.77	594.8 ± 48.04	417.1 ± 24.73<br>P=0.013*	493.9 ± 40.68
Liver TG (μmol/g)	91.56 ± 9.13	443.4 ± 31.98<br>P<0.0001a	289.7 ± 35.69<br>P=0.015*	295.8 ± 41.63<br>P=0.026*

ALT, alanine aminotransferase; AST, aspartate aminotransferase; TG, triacylglycerol; OCA, Obeticholic acid. NCD, normal chow diet (n=6); MCD-Vehicle (n=8); MCD-Alnustone (n=7); MCD-OCA (n=6). Data shown as the mean ± SEM. Statistical differences are denoted by a vs. NCD controls; * vs. MCD-vehicle; # vs. MCD-OCA. Two-tailed unpaired Student's *t* test for NCD vs. MCD-Vehicle and One-way ANOVA, with Tukey's multiple comparisons post-hoc test, NCD controls excluded.

Table S2. Clinical characteristics of normal control and MASLD subjects.

	Normal (n = 10)	MASLD (n = 10)	*P* value
Sex (Male/Female)	5/5	4/6	N/A
Age (years)	51.2 ± 14.08	54.5 ± 13.8	0.603
BMI (kg/m2)	23.1 ± 4.11	23.73 ± 4.41	0.745
Serum TG (mmol/L)	1.55 ± 0.54	1.24 ± 0.63	0.329
Serum TC (mmol/L)	5.11 ± 1.87	5.1 ± 1.12	0.994
NAS	1 (n = 6); 2 (n = 4)	3 (n = 5); 4 (n = 2); 5 (n = 3)	<0.001

BMI, body mass index; TG, triacylglycerol; TC, total cholesterol; NAS, non-alcoholic fatty liver disease activity score. Data are presented as mean ± SD. Significance is assessed by two-tailed unpaired Student's *t* test or rank sum test.

Table S3. Sequences of the primers used for RT-qPCR

**Gene**	**Species**	**Primer sequence (5' to 3')**
*Il1b*_forward	*Mus musculus*	CCGTGGACCTTCCAGGATGA
*Il1b*_reverse	*Mus musculus*	GGGAACGTCACACACCAGCA
*Tnfα*_forward	*Mus musculus*	CCTGTAGCCCACGTCGTAG
*Tnfα*_reverse	*Mus musculus*	GGGAGTAGACAAGGTACAACCC
*Adgre1*_forward	*Mus musculus*	CCCCAGTGTCCTTACAGAGTG
*Adgre1*_reverse	*Mus musculus*	GTGCCCAGAGTGGATGTCT
*Ccl2*_forward	*Mus musculus*	TACAAGAGGATCACCAGCAGC
*Ccl2*_reverse	*Mus musculus*	ACCTTAGGGCAGATGCAGTT
*Ccl5*_forward	*Mus musculus*	GCTGCTTTGCCTACCTCTCC
*Ccl5*_reverse	*Mus musculus*	TCGAGTGACAAACACGACTGC
*Col1a1*_forward	*Mus musculus*	TGCTAACGTGGTTCGTGACCGT
*Col1a1*_reverse	*Mus musculus*	ACATCTTGAGGTCGCGGCATGT
*αSMA*_forward	*Mus musculus*	CCCAGACATCAGGGAGTAATGG
*αSMA*_reverse	*Mus musculus*	TCTATCGGATACTTCAGCGTCA
*Cxcl10*_forward	*Mus musculus*	ATGACGGGCCAGTGAGAATG
*Cxcl10*_reverse	*Mus musculus*	ATGATCTCAACACGTGGGCA
*Tgfβ1*_forward	*Mus musculus*	CTCCCGTGGCTTCTAGTGC
*Tgfβ1*_reverse	*Mus musculus*	GCCTTAGTTTGGACAGGATCTG
*Ctgf*_forward	*Mus musculus*	TGACCCCTGCGACCCACA
Ctgf _reverse	*Mus musculus*	TACACCGACCCACCGAAGACACAG

*Calm1*_forward	*Mus musculus*	TGGGAATGGTTACATCAGTGC
*Calm1*_reverse	*Mus musculus*	CGCCATCAATATCTGCTTCTCT
*Calm2*_forward	*Mus musculus*	ACGGGGATGGGACAATAACAA
*Calm2*_reverse	*Mus musculus*	TGCTGCACTAATATAGCCATTGC
*Calm3*_forward	*Mus musculus*	GATGGCACCATTACCACCAAG
*Calm3*_reverse	*Mus musculus*	CGCTGTCTGTATCCTTCATCTTT
*β-Actin*_forward	*Mus musculus*	TGTTACCAACTGGGACGACA
*β-Actin*_reverse	*Mus musculus*	GGGGTGTTGAAGGTCTCAAA
*CALM1*_forward	*Homo sapiens*	TTGACTTCCCCGAATTTTTGACT
*CALM1*_reverse	*Homo sapiens*	GGAATGCCTCACGGATTTCTT
*CALM2*_forward	*Homo sapiens*	AGTGCTGCAGAACTTCGCCATG
*CALM2*_reverse	*Homo sapiens*	CAAGGTCTTCACTTTGCTGTCATC
*CALM3*_forward	*Homo sapiens*	GACCATTGACTTCCCGGAGTT
*CALM3*_reverse	*Homo sapiens*	GATGTAGCCATTCCCATCCTTG
*β-ACTIN*_forward	*Homo sapiens*	CATGTACGTTGCTATCCAGGC
*β-ACTIN*_reverse	*Homo sapiens*	CTCCTTAATGTCACGCACGAT
